# Gene duplications contribute to the overrepresentation of interactions between proteins of a similar age

**DOI:** 10.1186/1471-2148-12-99

**Published:** 2012-06-25

**Authors:** Like Fokkens, Paulien Hogeweg, Berend Snel

**Affiliations:** 1 Theoretical Biology and Bioinformatics, Department of Biology, Faculty of Science, Utrecht University, Padualaan 8, 3584CH, Utrecht, The Netherlands; 2 Netherlands Consortium for Systems Biology (NCSB), c/o NISB Bureau, University of Amsterdam, Science Park 904, 1098XH, Amsterdam, The Netherlands

**Keywords:** Network evolution, Protein age, Duplication, Subfunctionalization, Paralogs, Protein-protein interactions, Network growth model

## Abstract

**Background:**

The study of biological networks and how they have evolved is fundamental to our understanding of the cell. By investigating how proteins of different ages are connected in the protein interaction network, one can infer how that network has expanded in evolution, without the need for explicit reconstruction of ancestral networks. Studies that implement this approach show that proteins are often connected to proteins of a similar age, suggesting a simultaneous emergence of interacting proteins. There are several theories explaining this phenomenon, but despite the importance of gene duplication in genome evolution, none consider protein family dynamics as a contributing factor.

**Results:**

In an *S. cerevisiae* protein interaction network we investigate to what extent edges that arise from duplication events contribute to the observed tendency to interact with proteins of a similar age. We find that part of this tendency is explained by interactions between paralogs. Age is usually defined on the level of protein families, rather than individual proteins, hence paralogs have the same age. The major contribution however, is from interaction partners that are shared between paralogs. These interactions have most likely been conserved after a duplication event. To investigate to what extent a nearly neutral process of network growth can explain these results, we adjust a well-studied network growth model to incorporate protein families. Our model shows that the number of edges between paralogs can be amplified by subsequent duplication events, thus explaining the overrepresentation of interparalog edges in the data. The fact that interaction partners shared by paralogs are often of the same age as the paralogs does not arise naturally from our model and needs further investigation.

**Conclusion:**

We amend previous theories that explain why proteins of a similar age prefer to interact by demonstrating that this observation can be partially explained by gene duplication events. There is an ongoing debate on whether the protein interaction network is predominantly shaped by duplication and subfunctionalization or whether network rewiring is most important. Our analyses of *S. cerevisiae* protein interaction networks demonstrate that duplications have influenced at least one property of the protein interaction network: how proteins of different ages are connected.

## Background

The wealth of sequence data from a wide range of species, has allowed for large-scale studies of genome evolution and detailed reconstruction of the ‘parts lists’ of our earliest ancestors [1,2]. The study of network evolution not only requires these detailed ‘parts lists’, but also information on how these parts are assembled into a molecular machinery in different organisms. Despite the progress in both the generation of large-scale functional data in multiple organisms, as well as the inference of functional relations from sequence data, the overlap in functional networks in different species is typically very small. The reconstruction of ancestral networks on a scale that would allow for general statements on network evolution is not yet possible [3].

Previous studies attempt to circumvent this problem by assigning an age to proteins in an *S. cerevisiae* protein interaction network, assuming that patterns of connectivity between proteins of different ages offer a glimpse on how the network changed over time. A recurrent observation in these studies is the simultaneous emergence of interacting proteins [4-6]. To date, two distinct theories have been put forward to explain this phenomenon. Multiple interacting proteins, added to the network at the same time, may be more likely to be functional and therefore under positive selection [4]. Alternatively, a tendency to interact with proteins of similar age can arise as a side effect of a neutral network expansion process in which new proteins are added to network peripheries while old proteins are mainly located at network cores [5]. In this work, we amend both these explanations by demonstrating that gene duplication events contribute to the overrepresentation of interactions between proteins of similar age.

Protein age, as defined by the taxonomic distribution of the family it belongs to, is assumed to correspond to a time frame in which the protein was ‘added’ to the network [4-6]. However, few genes in *S. cerevisiae’*s genome and thus in its protein interaction network have emerged absolutely de novo. Most genes are the result of either small scale or whole genome duplications, replacing an ancestral gene by two daughter genes.

In the classical view of functional divergence after gene duplication, one of the daughters keeps the ancestral function while the other is free to evolve an entirely new function (neofunctionalization) [7]. On the network level, this would indeed correspond to a node being ‘added’ to the network (namely the node evolving a new function), but the protein evolving a new function, unless it is not recognized as a homolog, belongs to the same family as its paralog and thus has the same ‘age’ by definition. Thus, even if network evolution can be considered as a process in which new nodes are simply ‘added’ to the network, the age of a protein does not correspond to the time frame of emergence in the network.

Moreover, neofunctionalization is not the only possible scenario of divergence after duplication [8-13]. For example, duplicate genes are preserved in the genome to achieve a dosage increase [14] or daughter genes both perform part of the ancestral function [15] (subfunctionalization). These processes cannot be modeled by ‘adding’ proteins to a network. If, due to network rewiring, genome and network evolution would be completely independent, we would expect paralogs to behave like random pairs in the network. On the other hand, if gene duplication events leave an imprint on the network, we would expect paralogs to share more interactions partners than non-paralogs, reminiscent of their initial complete redundancy. Indeed, even if the vast majority of paralog pairs does not share any interaction partners, the relative overlap in interaction partners of paralogs is higher than of pairs belonging to different families [16,17].

Here, we investigate the influence of gene duplication events on the age structure of *S. cerevisiae* protein interaction networks. We find that interparalog interactions account for a small part of the overrepresentation of interactions between proteins of a similar age. Intriguingly, we find another, unexpected effect of gene duplications on the age structure of the network. It turns out that the major contribution to the observation that proteins interact with proteins of a similar age is from interaction partners that are shared by paralogs, mostly likely an ancestral interaction that is preserved after duplication. We investigate whether this result can occur as a side effect of neutral network growth by duplication and divergence, and find that our simple model can only replicate an overrepresentation of interparalog edges, not the conservation of edges with proteins of the same age after duplication.

## Results and discussion

**Figure 1:**
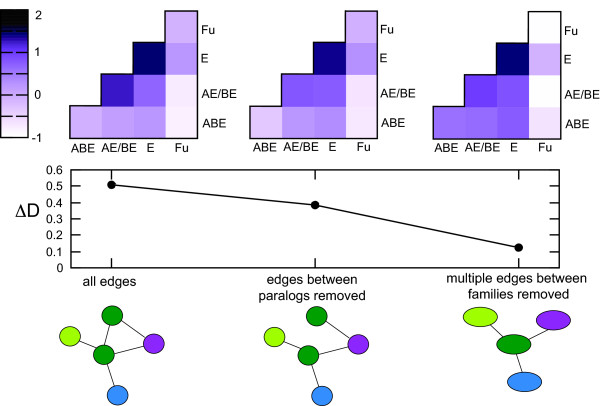
**ΔD and interaction densities between age groups in the original and collapsed protein interaction network.** We calculate the ΔD value and the normalized interaction densities for an *S. cerevisiae* literature curated network, using the taxonomic distribution of EggNOG orthologous groups to determine protein age. Round network nodes correspond to proteins whereas oval network nodes correspond to protein families. Different colors indicate different families. If we remove all edges between paralogs, the ΔD value decreases and the normalized interaction densities for this network show that the strongest effect is in interactions between proteins of age AE/BE. It turns out that mainly interactions between homologous components of the Spliceosome have been removed in this age category. We then continue to remove all edges that are redundant on a protein family level, thus collapsing the network into a network where nodes are no longer proteins, but protein families. We show that ΔD is decreased dramatically in this network. There is neither a specific family nor age group overrepresented among the nodes that have edges removed.

We perform an in depth analysis on the effect of gene duplications on age structure in an *S. cerevisiae* literature curated protein-protein interaction network (PIN) [18], consisting of 3268 nodes and 12058 edges. We assign an age to the 2476 nodes that belong to a known protein family [19], based on the taxonomic distribution of this family. We use the work by Kim and Marcotte as an anchor point and group proteins into the same 4 age categories they use, ranging from families that have members from all three kingdoms (Archaea, Bacteria and Eukaryotes, named ABE), those with members from only two kingdoms (AE/BE) to Eukaryote- (E) and Fungal- (Fu) specific families (E) [5]. Moreover, we use their method to calculate normalized interaction densities between different age groups and implement the statistic they propose, ΔD, to measure age-dependence among these interaction densities (see Materials and Methods and [5] for further detail). A positive value of ΔD indicates a higher than expected connectivity between proteins of similar age categories. The literature curated PIN has a ΔD value of 0.51 (Figure 1). In addition to ΔD we define a new measure to quantify interaction densities between age groups and the potential gradient in these densities. Results using this alternative measure ΔD_new_ are discussed in the last section of the results and discussion.

### No evidence for artifacts in the data causing the observed interaction preference among proteins

The tendency to interact with proteins of a similar age has been reported by several independent studies, each using a different PIN, different families to infer age and different levels of granularity in age categories. However, we need to be as sure as possible that this phenomenon is not caused by any artifacts in the data. To correct for possible biases in the literature curated PIN, we do the same analyses on 3 other networks, one based on Y2H [20], one on TAP/MS [21-23] data and a combination of both techniques (HTP network from [5], see Materials and Methods for more detail), and find ΔD values ranging from 0.48 to 0.63 (Additional file 1: Table S1). The relatively small overlap in interactions of these networks (Additional file 1: Figure S2) indicates they sample different portions of the underlying real PIN [20], though of course some of the interactions that occur in only a single network are False Positives. Interactions between abundant proteins are likely to be overrepresented in all of these networks [24]. We compare the abundance of proteins in the different PINs to a background distribution of all proteins for which abundance was measured (Additional file 1: Table S3, data obtained from [25]). We find that only networks including interactions based on TAP/MS data differ significantly from the background. To ensure the interaction preference among proteins of a similar age is not limited to abundant proteins and thus not representative of the underlying complete interaction network, we remove the 10, 50, 100, 500 and 1000 most abundant proteins and recalculate ΔD. We find that removal of the most abundant proteins does not lead to a decrease in ΔD (Additional file 1: Table S4) and conclude that interaction preference among proteins of a similar age is not limited to abundant proteins. Similarly, we determine which functional categories assigned to protein families are overrepresented in the different networks, remove all proteins from the categories and find again that ΔD does not decrease (Additional file 1: Table S5).

We experiment using different age groups representing various other intervals on the species tree and find that ΔD does not depend on the specific age categories ABE, AE/BE, E and Fu (Additional file 1: Table S6). Because our definition of age is dependent on the taxonomic distribution of a protein family, we expect that slowly evolving protein families, as their members are recognized across more distant species, tend to be older [26]. Indeed, if we compare the distribution of Dn/Ds ratios [27] among the different age categories, we find faster sequence evolution for young proteins (Additional file 1: Figure S7). Interacting proteins are under similar evolutionary constraints and tend to have similar rates of evolution [28-30], thus the overrepresentation of interactions between proteins of a similar age could be a side effect of the correlation of protein age with evolutionary rate. If the observed ΔD value would depend on similar rates of sequence evolution rather than on similar age, we would expect that if we bin proteins according to their Dn/Ds ratio (as if this was their age), ΔD for these categories would exceed ΔD based on age groups. In contrast, we find that if we calculate ΔD based on evolutionary rate, it is −0.05 while for this network (a subnetwork of the original network as not all proteins in the network are assigned a Dn/Ds ratio), ΔD based on age groups is 0.54 (Additional file 1: Table S8). Even though protein age correlates with the rate of sequence evolution, the latter is not the determining factor in the interaction preference among proteins of a similar age. In conclusion, we have found no evidence that a positive ΔD value is caused by biases in the data.

### Interactions between paralogs play a minor role in the interaction preference among proteins of a similar age

Several studies investigating the evolution of protein complexes revealed that they often originate from duplications of genes encoding self-interacting proteins [31-35]. On a network level, this would result in clusters of interacting proteins of the same age (Additional file 1: Figure S9). Interparalog interactions are thus a possible explanation for the ‘simultaneous emergence’ of interacting proteins. Of the 7210 interactions between proteins that belong to a known family, 430 are interactions between paralogs (~ 6%), belonging to 107 different families (Additional file 1: Table S10). Of these 430 interparalog edges, 258 are interactions between members of the same protein complex (~ 60%).

Even though they comprise only a small fraction of all the edges in the PIN, interactions between paralogs are more abundant than one would expect given the size distribution of protein families and even the age structure of the network (P < 10^-4^, 100000 random redistributions of family labels over nodes. Family labels are only shuffled within the same age category to preserve ΔD). If we remove all interparalog edges from the network, we reduce the network to 3228 nodes and 11628 edges (in the original network, 40 proteins interact only with family members. In this reduced network they have no edges, and therefore they are removed) and ΔD decreases with approximately 24% to a value of 0.38 (Figure 1). This value of ΔD is still significantly higher than random (P < 10^-4^, randomization by redistributing family-labels over the network without interparalog edges 100000 times), indicating that growth of functional modules (e.g. protein complexes) by duplication of subunits only accounts for part of the overrepresentation of interactions between proteins of a similar age.

### Interaction partners *shared by* paralogs play a major role in the interaction preference among proteins of a similar age

Gene duplications do not only influence the PIN by generating interactions between paralogs. Ancestral interactions with other proteins, if conserved in both paralogs after duplication, can also alter network topology. In this specific literature curated PIN, the relative overlap of interaction partners between paralogs is significantly higher than of pairs belonging to different families (P ~ 0.0, Additional file 1: Table S11). This overlap does not necessarily affect the age structure of the network. Interestingly, we find that the interaction partners shared by paralogs are more often of the same age as the paralogs, than the interaction partners they don’t share (P < 4.6e-17, Additional file 1: Table S12), indicating that duplication of protein interactions can also contribute to a positive ΔD.

**Figure 2:**
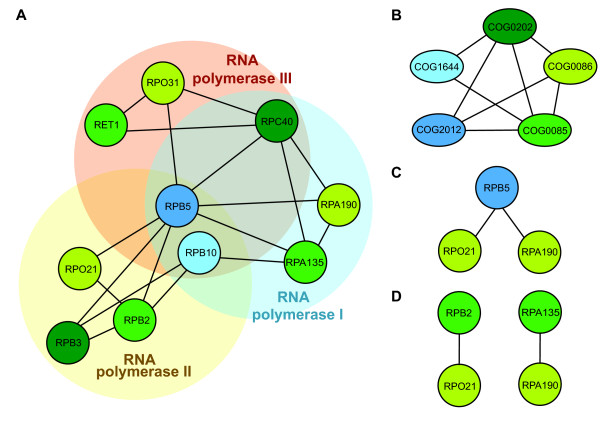
**Example of network collapse into protein families.** We show here part of the Literature Curated *S. cerevisiae* protein interaction network, involving interactions between certain components of RNA polymerases. **A**. The sub network in its original state. Nodes are colored according to their age (green: ABE, blue: AE/BE), individual families are in different shades of the same color. **B**. The subnetwork collapsed into protein families. **C**. Duplication in a single family: two edges that share a node (RBP5). **D**. Duplications in both families: non-overlapping edges (RPB2-RPO21, RPA135-RPA190, RET1-RPO31).

In order to reduce the effect of interaction conservation after duplication events on ΔD, we collapse the network into connected families (see Figure 2 for an example of collapsing network of interacting proteins into a network of interacting families). Interestingly, ΔD decreases to 0.12, a value that is not significantly different from random (P ~ 0.1, randomization by redistributing family-labels over the collapsed network 100000 times, Figure 1, similar results in other PINs: Additional file 1: Table S1). In addition to intrafamily edges, we have removed all edges that occur multiple times between family pairs (Additional file 1: Table S13). The decrease in ΔD shows that families of a similar age often have multiple edges connecting their members.

The most likely scenario (requiring the smallest number of evolutionary events) in which gene duplication generates additional edges between two families, is when a member *A* of one family duplicates and both daughters *A’* and *A”* keep the ancestral interaction with the protein *B* from the other family. The two edges representing these interactions overlap as both contain the protein *B*. For example, RPB5 (COG2012) and RPB10 (COG1644) are RNA polymerase subunits common to all three polymerases and are connected to different members of COG0085 and COG0086 (Figure 2C). On the other hand, if proteins from both families duplicate, the edges representing the interactions do not necessarily overlap: e.g. if *A’* interacts with *B’* and *A”* interacts with *B”* and *A’* does not interact with *B”* and *A”* does not interact with *B’*. This scenario occurs in the RNA polymerases as well: proteins RPA135, RPB2 and RET1, members of COG0085, are connected to RPA190, RPO23 and RPO31 respectively, members of COG0086, and part of RNA Polymerase I, II and III [36-38] (Figure 2D).

For each family-pair that occurs multiple times in the network (i.e. multiple edges exist between members of these families), we calculate the fraction of protein-pairs that is overlapping. We find that for 80% of the families, all protein-pairs overlap (*A’-B* and *A”-B*, Additional file 1: Table S14), suggesting that the amplification of the number of interactions between proteins of a similar age occurs mainly through asymmetric expansion rather than duplication and reuse of small functional modules. Interestingly though, if both families are of the same age, this fraction is much lower (65%). However, there is a strong bias towards pairs of old families, suggesting gradual duplication of functional modules (given more time, duplication of additional subunits is more likely), rather than duplication of entire functional modules at a time [33].

### Age-dependent interaction densities in an extended Duplication-Divergence model

The results described above demonstrate that gene duplications contribute strongly to the observed interaction preference among proteins of a similar age. First of all, interparalog edges explain part of the overrepresentation of edges among proteins of a similar age in the network, suggesting a role for functional module growth by duplication of subunits. The major contribution in most networks however, is from the conservation of ancestral interactions with proteins of a similar age. Are these interactions *preferentially* conserved? In other words, if the ancestral protein interacted with some proteins that are older and some proteins that are of the same age, do the daughter genes after duplication typically lose the interactions with the older proteins and do they tend to keep those interactions with the proteins of the same age? Or is a small bias in the number of interactions with proteins of a similar age of the ancestral protein, amplified by subsequent duplication events? In other words, does natural selection play a role or does this phenomenon arise as a side effect of network growth by duplication and divergence?

Due to limited availability in protein interaction data in different species, the direct inference of ancestral protein interaction networks and subsequent evolutionary events is primarily anecdotal [3]. Therefore we prefer to use a network growth model to directly test some of our assumptions on network evolution. First and foremost, we want to establish whether conservation of interactions with proteins of a similar age after duplication, arises as a trivial side effect of neutral network growth. We adjust a well-studied and simple model of network growth by node duplication [39], which we will refer to as the Duplication-Divergence (DD) model, to accommodate family relations between nodes. In the model, we use families to define the age of a node and to calculate statistics on paralogs in order to compare them to those obtained from the data.

The model is initialized with a fully connected graph of 4 nodes, which are of 4 different families but have the same age. When a randomly selected node is duplicated, both copies are connected to the same nodes to which the ancestral node was connected. Duplication is followed directly by a rapid subfuctionalization process: for each ancestral neighbor, we delete its edge with one of the two daughter nodes with a probability *q*. During the subfunctionalization steps, it is possible to favor one of the two daughter nodes when deleting an edge (parameter *s*), leading to systematic asymmetric divergence [40-42]. In our extension of the original model, with a probability *a,* this subfunctionalization process is accompanied by drastic changes in sequence, leading to one of the paralogs founding a new family (i.e. not recognizable as a paralog). Otherwise, both paralogs belong to the same family and thus have the same age. With a probability *p* a new connection is formed between the duplicates, analogous to e.g. a homodimer becoming a heterodimer (see Materials and Methods and Additional file 1: Figure S15 for more detail).

Previously published results show that a DD model without the implementation of protein families can only yield networks with a negative ΔD value, i.e. networks in which nodes mostly interact with nodes of a different age [5]. This is because in the DD model, nodes with a high degree have a larger probability to connect to a new node, by duplication of one of their neighbors. Since in network growth models in general, old nodes have more neighbors than young nodes (simply because they have had more time to gain edges) this results in old nodes preferably gaining a new edge. In a model without family relations between nodes, one of the twin nodes will always be assigned a new age after duplication and therefore the edges gained will be mainly connecting an old node to a young node.

**Figure 3:**
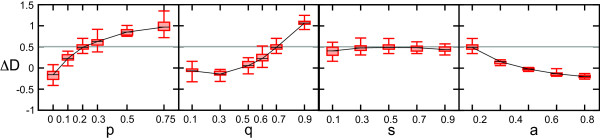
**ΔD values for the extended DD model under different parameter conditions.** Increase in values of *p* and *q* and decrease of *a* lead to higher ΔD values in the network, divergence symmetry *s* has very little effect. Default parameter conditions are **p** = 0.2, **q** = 0.7, **s** = 0.5, **a** = 0.2, each plot shows ΔD values when one of these parameters is varied while the others are kept at default values (for full parameter sweeps we refer to Figure S16). The gray line is the ΔD value of the yeast LC PIN. Boxes show the .25 and .75 percentile of 20 runs, the error bars show the extreme values and the black line is the mean of 20 runs.

Using our implementation of protein age, that is more congruent with the bioinformatic data analysis, we systematically study the DD model by running it under many different parameter conditions. We find that in our extended model a positive ΔD value is possible under parameter conditions that have been shown to yield networks that are topologically similar to yeast protein interaction networks [5]. Given a low probability of founding a new family (parameter *a*), a high level of divergence after duplication (parameter *q*) and a relatively high probability of a connection between twin nodes after duplication (parameter *p*) our model yields networks with a ΔD value that is comparable to that of a yeast PIN (Figure 3, Additional file 1: Figure S16).

**Figure 4:**
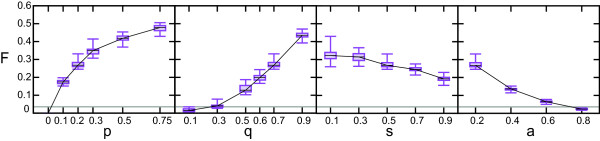
**The fraction of interparalog edges for the extended DD model under different parameter conditions.** As is the case for ΔD values, an increase in values of *p* and *q* and decrease of *a* lead to higher fraction of edges that connect paralogs in the network, divergence symmetry *s* has very little effect. Note that low values of *p* already have a large impact on the fraction of edges connecting paralogs: with p = 0.2 and a = 0.2, the probability of gaining an edge between daughter nodes after a duplication event in the network is p*(1-*a*) = 0.16, yet this small probability of an edge gain between daughter nodes after duplication leads to ~30% of all edges connecting paralogs. Default parameter conditions are **p **= 0.2, **q** = 0.7, **s** = 0.5, **a** = 0.2, each plot shows the fraction of interparalog edges when one of these parameters is varied while the others are kept at default values (for full parameter sweeps we refer to Figure S18). The gray line is the fraction of edges that connect paralogs of the yeast LC PIN. Boxes show the .25 and .75 percentile of 20 runs, the error bars show the extreme values and the black line is the mean of 20 runs.

These specific parameter conditions lead a high number of interparalog interactions in the network: due to high divergence after duplication the relative contribution of novel edges between twin nodes is higher (Figure 4, Additional file 1: Figure S17). In the DD model the positive ΔD value hinges on interactions between members of the same family, as is also illustrated by the high interaction densities between proteins of the exact same age (Additional file 1: Figure S18). If we remove interparalog edges from a model network, ΔD decreases below zero and if we collapse the model networks into networks of protein families, ΔD decreases even further (Additional file 1: Figure S19). In the data we do not observe such a preference for young families to interact with old families.

We gain two important insights from the extended DD model. First of all, we find that the number of interparalog edges in networks produced by the model is much higher than one might expect based on the values of *p* and *a* alone. It turns out that only a small fraction (0-2%, depending on parameter conditions) of interparalog edges in the model stem directly from the gain of an interaction between two daughter nodes immediately after a duplication event. After a duplication event in which an edge is gained between daughter nodes, this new edge can be propagated in the network through subsequent duplication of these daughter nodes. Importantly, this demonstrates that the effect of relatively rare events on network topology can be amplified in networks that grow by duplication and subfunctionalization of nodes. Moreover, this mechanism indicates that previous estimates of the degree of neofunctionalization after duplication that are based on the overrepresentation of interactions between paralogs are likely to be too high [43,44]. Secondly, despite the fact that conservation of ancestral interactions is more likely to occur under these parameter conditions (Additional file 1: Figure S20), we find that low levels of functional divergence alone do not lead to a higher ΔD value (Figure 3, Additional file 1: Figure S21). This indicates that an overrepresentation of edges between proteins of a similar age, due to conservation of ancestral interactions in both duplicates, does not arise automatically from a process of network growth by node duplication such as we modeled here.

### Alternative measures for age-dependence in interaction densities

**Figure 5:**
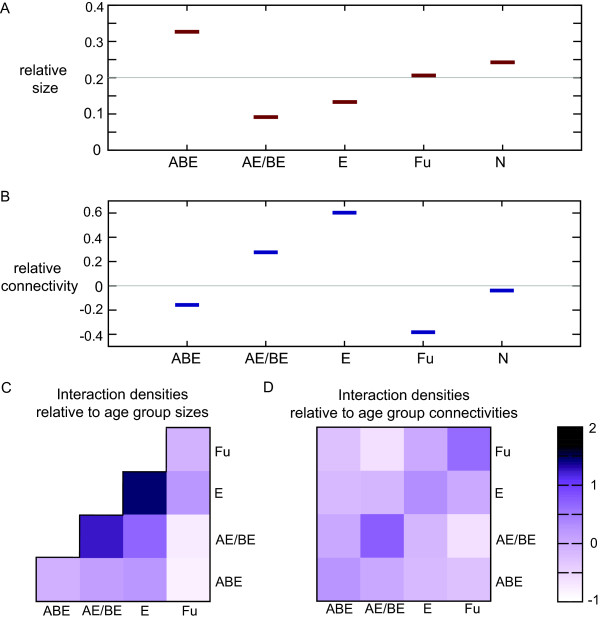
**Interaction densities depend on size and connectivity of age groups.** Interaction densities depend on age group sizes: the connectivity between age groups is normalized by the maximum possible connectivity: the number of connections if all members from the age groups would be interacting. This density is then again normalized by the interaction density of the entire network to allow for comparison between networks of different edges densities (see Materials and Methods for more detail). Age groups that are small and/or have low general connectivity will have low interaction densities with any age group. Interaction densities thus do not only represent a relative connectivity between a pair of age groups (or within one age group), it also reflects the overall connectivity as well as the size for each individual age group. **A**. Relative size for each age group in the LC network: the proportion of nodes that belong to this age group. The grey line denotes the relative size if all age groups would be of the same size. ABE and Fu are the largest groups. N is the group of proteins that are not assigned to an eggNOG family. **B**. Relative connectivity for each age group in the LC network, calculated as follows: log2 (avg(degree_age group_) / avg(degree_network_)). The grey line denotes the relative connectivity if it would have been the same for all age groups. Age groups ABE and Fu have a relatively low degree. N is the group of proteins that are not assigned to an eggNOG family. **C**. Interaction densities between age groups in the LC network: age groups ABE and Fu have in general low interaction densities with each age group reflecting their large size and low connectivity rather than a specific relation between two age groups. D. An improved method of calculating interaction densities: connectivity normalized by expected connectivity (see Materials and Methods for more detail). These densities are independent of the age groups sizes or degree and represent only a specific property of a pair of age groups. Our alternative measure ΔD_new_ is calculated based on these interaction densities.

In [5] the number of interactions between members of two age groups is normalized with respect to the number of nodes in each age group (representing the maximum number of edges that is possible between these age groups (see Materials and Methods)). As a consequence, age groups with low connectivity in general have lower interaction densities (Figure 5). Moreover, ΔD is sensitive to random removal of nodes or edges from the network: it declines as more nodes or edges are removed (Additional file 1: Figure S22), while random removal of nodes and edges should not affect the overall tendency to interact with nodes of a similar age in the network.

We define an alternative measure for the tendency to interact with proteins of a similar age, ΔD_new_, based on interaction densities normalized by the age groups’ connectivity (see Materials and Methods for more detail). This new measure neither reflects differences in connectivity for different age groups (Figure 5) nor does it scale with the number of nodes or edges in the network (Additional file 1: Figure S22). We reperform all of our analyses using ΔD_new_ instead of ΔD. We find ΔD_new_ values ranging from 0.35 to 0.56 (Additional file 1: Table S1) indicating that the interaction preference among proteins of similar age is neither due to artifacts in the measure of interaction density nor to the measure of the gradient in interaction densities. We test how ΔD_new_ depends on possible biases in the data, such as protein abundance, overrepresented functional categories, evolutionary rate and the choice of age groups and find that, like ΔD, these biases do not affect the positive value of ΔD_new_ (Additional file 1: Table S4, Additional file 1: Table S5, Additional file 1: Table S6 and Additional file 1: Table S8).

If we remove interparalog edges we find that ΔD_new_ is decreased for all networks (Additional file 1: Table S1, Additional file 1: Figure S23). If we collapse our networks into networks of protein families (Figure 1), we find that ΔD_new_ decreases in 3 out of 4 networks (Additional file 1: Table S1, Additional file 1: Figure S23). If we compare the ΔD_new_ values to those of randomized networks (randomization by redistributing family labels over the network), we find that ΔD_new_ is not significantly different from random networks for two out of 4 networks: the Y2H and the TAP network (Additional file 1: Table S1). In the model networks, there is little difference between ΔD_new_ and ΔD (Additional file 1: Figure S24). In conclusion, we have factored out age group properties confounding the previous definition of interaction densities, namely size and connectivity, and find that our results remain largely unchanged.

## Conclusion

Several studies relate the age of a protein to how that protein is embedded in the molecular machinery [6,45-49]. In order to use this information to understand the evolution of the molecular machinery, one needs a clear conception of what ‘age’ actually is. Concerns regarding potential biases in protein age defined through the taxonomic distribution of detected homologs have been raised before [26,50]. This is important because an incorrect understanding of protein age can lead to premature conclusions on network evolution. For example, the observation that old proteins tend to have more interactions has been proposed as evidence supporting the Preferential Attachment model of network evolution [49,51], but a slow rate of sequence evolution as well as a low propensity for gene loss have been associated with increased connectivity [27,52-54], which would be an alternative explanation.

We test whether the overrepresentation of interactions between proteins of a similar age can be explained by biases in both the genomic as well as the functional data and find that this is not the case. In contrast, interactions between paralogs as well as interaction partners shared by paralogs account for part of the tendency to interact with proteins of a similar age. The fact that interaction partners that are shared by paralogs are more often of the same age has not been previously reported. The most parsimonious evolutionary scenario explaining the fact that two paralogs share an interaction partner, is one in which the pre-duplication ancestor of the two paralogs had an interaction with another protein and this ancestral interaction was conserved in both daughter nodes after duplication. An initial small bias to interact with proteins of a similar age could have been amplified by duplication events.

We test this hypothesis in a network growth model, which is initialized with a fully connected network of 4 nodes of the same age. We find evidence that amplification through duplication is possible in the case of interparalog edges: novel edges between paralogs are created at a low rate but because of subsequent duplications of these interactions, creating these novel edges can have a profound effect on network topology. If duplication and the conservation of ancestral interactions with proteins of a similar age would be sufficient to generate an interaction preference among proteins of a similar age we expect it to emerge from this model. The fact that our model can only explain the part of the interaction preference among proteins of a similar age that is caused by interacting paralogs, suggests that future work should be directed at identification of additional important factors. For example, our model neither implements de novo gene invention or interaction gain, network rewiring, nor gene loss. Moreover, protein interaction networks tend to include several types of interactions, ranging from phosphorylation to possibly indirect interactions of proteins that belong to the same complex. In summary: our analyses of protein interaction data suggest an important role for gene duplications in the preference to interact with proteins of a similar age. Yet results from our model indicate that a process of duplication and subfunctionalization alone does not explain the preference to interact with proteins of a similar age we observe in *S. cerevisiae* protein interaction networks.

## Methods

### Protein families and protein age in protein interaction networks

The literature curated (LC) network and the network based on Y2H data combined with TAP/MS data (HTP network) were taken from the Supplementary Material of the paper by Kim and Marcotte [5]. The literature-curated network was based on data from BioGRID [18]. Interactions that were only supported by high throughput data were removed, as well as all protein-RNA interactions, interactions supported only by co-localization or co-fractionation or data from [21,55,56] were excluded. The HTP network was created by compiling data from [22,23,57-60], including only those interactions that have been supported by more than one study (studies [22] and [59] were counted as one). From both the LC and the HTP network ribosomal proteins were excluded (see original paper [5] for more detail). The network based on Y2H data [20] was downloaded from interactome.dfci.harvard.edu/S_cerevisiae/download/Y2H_union.txt. We construct a binary TAP/MS network by using PE scores calculated by [21] based on data from [22,23] and a PE score cutoff of 0.2. PE scores were downloaded from http://interactome-cmp.ucsf.edu/.

We want to investigate the effect of gene duplications on the tendency to interact with proteins of a similar age and to avoid unnecessary complications we use protein families (as in [4,6]) rather than domain families (as in [5]) to define the age of a protein. We download EggNOG orthologous groups [19] (COG. NOG) from ftp://eggnog.embl.de/eggNOG/2.0/ and assign an age to each group based on the species distribution in this group. EggNOG uses NCBI taxonomy identifiers for its species, we use NCBI taxonomy (nodes.dmp in taxdump.tar.gz, downloaded from ftp://ftp.ncbi.nih.gov/pub/taxonomy/) to determine which internal node in the species tree provided by EggNOG corresponds to the ancestor of e.g. all Bacteria, gathered all leaves that were under that internal node and scanned the EggNOG families for the presence of species with these NCBI taxonomy identifiers. Two identifiers have changed in NCBI: 5058 in EggNOG is 746128 in NCBI and 382253 in EggNOG is 434922 in NCBI. If a group only contains fungal proteins, it was assumed to have been invented in Fungi and was assigned the age Fu, if it consists of Eukaryotes only it was assigned the age E. If a group contains at least one protein from either Bacteria or Archaea it was assumed to have emerged in the ancestor shared by Archaea and Eukaryotes or in the First Eukaryotic Common Ancestor (assuming that proteins that are present in Bacteria and Eukaryotes only result from an endosymbiosis event leading to the mitochondrion) and was assigned the age AE/BE. If a group contains at least one protein from Bacteria and at least one from Archaea it was assumed to have been present in Last Universal Common Ancestor and was assigned the age ABE. If we implement age categories based on other intervals on the species tree, ΔD range between 0.6 and 0.79 (Additional file 1: Table S6).

All data (e.g. abundance, age, complex membership, etc.) on individual proteins used in this study is provided in Additional file 1: Table S25. For complex membership, we use the list of yeast proteins assigned to different GO macromolecular complexes obtained from http://www.yeastgenome.org/cgi-bin/GO/goSlimMapper.pl.

### Interaction preference with proteins of a similar age: ΔD and ΔD_new_

We use the metric described in [5] for each pair of age groups *m* and *n*, the normalized interaction density is calculated as follows:

(1) $$D_{m,n}=\log_{2}\frac{I_{m,n}/E_{m,n}}{2L/(N(N-1))}$$

Where

I_*m,n*_ = the number of edges observed between age groups *m* and *n*

E_*m,n*_ = the maximum number of edges that is possible between age groups *m* and *n*, and is calculated as follows:

Within an age group: $E_{n,n}=N_{n}(N_{n}-1)/2$

Between different age groups: $E_{m,n}=N_{m}\times N_{n}$.

Where

N_*m*_*,* N_*n*_ = the number of nodes with age *m*, *n* respectively.

N = the total number of nodes

L = the total number of edges

To compare these interaction densities, we calculate the average interaction density gradient ΔD.

(2) $$\Delta D=\frac{\sum_{n=2}^{G}\sum_{m<n}\left(D_{m+1,n}-D_{m,n}\right)}{G(G-1)/2}$$

Where G is the number of age groups (4 in this study). These equations are equal to those in the original paper by Kim and Marcotte [5].

The interaction densities, as calculated by Kim and Marcotte, are normalized with respect to the number of nodes in the age groups. However, the connectivity differs quite strongly per age group. For example, the fungal specific proteins are not as densely connected as older proteins hence interaction densities between the Fu age group and all other age groups are typically low (Figure 1). If we want a low density to correspond to an underrepresentation of interactions between two specific age groups we should consider normalizing by the number of interactions we would expect based on the *connectivity* of the two age groups rather than their size. We therefore define alternative interaction densities in which we divide the frequency of observing an interaction between proteins of age group X and age group Y by the expected frequency of observing this interaction. For example, the LC network has 12058 edges, 7210 of which are between proteins that are assigned to a protein family and thus have an age. This corresponds to 2*7210 = 14420 ‘edge ends’, of which 5561 are occupied by a protein of age ‘ABE’: there are 1256 edges between two proteins that both have age ‘ABE’ and 3049 edges between a protein of age ‘ABE’ and a protein of a different age (2*1256 + 3049 = 5561). The observed frequency of edges between proteins that both belong to age group ‘ABE’ equals 2512/14420 ~ 0.174, while the expected frequency equals (5561/14420)^2^ ~ 0.149. The normalized interaction density between ‘ABE’ and ‘ABE’ is log_2_(0.174/0.149) = 0.23.

The original measure ΔD was calculated based on only part of the differences in densities between pairs of age groups. For our measure ΔD_new_ we use all the differences between pairs of age groups to quantify the gradient in our new set of interaction densities:

(3) $$\Delta D=\frac{\sum_{n=2}^{G}\sum_{m<n}^{G-1}\left(D_{m+1,n}-D_{m,n}\right)+\sum_{n=1}^{G-1}\sum_{m>n}^{G}\left(D_{n,m-1}-D_{n,m}\right)}{G^{2}}$$

Where G is the number of age groups (4 in this study) and D_m,n_ is the interaction density between age groups m and n normalized by the expected interaction density as described above.

### Network growth model

We implement the extended Duplication Divergence model using the Igraph package to represent graphs. We initialize the model with a fully connected graph consisting of 4 nodes. The seed graph does affect network topology in the DD model [61], we choose a seed graph similar to the one used in [5]. We want to focus on the effect of implementing protein families rather than other topological characteristics such as for example the shape of the degree distribution. If the DD model is initialized with this graph it can produce networks with topological characteristics similar to *S. cerevisiae* PINs [5]. The nodes in this seed graph all belong to different families, but these families do have the same age. We initialize the families and ages in the model as such because we want to test whether an initial interaction preference for proteins of a similar age will be amplified through a process of duplication and subfunctionalization.

At the end of a model run, when the network reached its target size of 3000 nodes, we group the different ages into 4 different groups, trying to keep the 4 groups of approximately the same size, if possible, to avoid large variance in ΔD due to sparsely populated age groups. Keeping the sizes of the age groups similar to those observed in the data has little effect on either ΔD or ΔD_new_ (Additional file 1: Figure S26). In the data ~20% of proteins have no age, leading to a lower fraction of edges that connect paralogs. We randomly select 600 nodes from the model network and designate them as nodes without an age in order to rule this out as the major contributor to the difference in the percentage of connected paralogs. We find that ΔD remains very similar and that the fraction of edges that connect paralogs is decreased but still a lot higher than in the data (Additional file 1: Figure S27).

Each iteration a random node *X* is selected and duplicated with all of its edges, resulting in nodes *A* and *B*. If a random number between 0 and 1, is lower than *a*, daughter node *A* (identical to *B*) is assigned a new age, while *B* still has the ancestral age (we assume one node needs to perform part of the ancestral function). Then, for each interaction partner *Y* of *A* and *B*, if a random number between 0 and 1 is lower than *q* the interaction between either *A* and *Y* or *B* and *Y* is deleted. If a random number is lower than s, we delete the interaction between *A* and *Y*, otherwise we delete the interaction between *B* and *Y*. This means that if s > 0.5, the interaction with the daughter node that can be assigned a new age is more likely to be deleted (the node that diverges faster in sequence, also loses more interactions). Finally, we draw a random number and if this number is lower than *p*, we create a new edge between A and B.

During the subfunctionalization process, it is possible for a node to lose all of its edges. In this case the node will be deleted and the network remains unchanged except for the fact that the node that ‘duplicated’ may have a new age.

## Abbreviations

PIN, Protein Interaction Network; DD, Duplication-Divergence; LUCA, Last Universal Common Ancestor; LECA, Last Eukaryotic Common Ancestor; GO, Gene Ontology; LC, Literature Curated; HTTP, High Throughput; Y2H, Yeast two-hybrid; TAP/MS, Tandem Affinity Purification, followed by Mass Spectrometry.

## Competing interests

The authors declare no competing interests.

## Authors’ contributions

PH and BS conceived of the study and assisted in writing the manuscript, LF participated in the design of the study, performed the analyses and wrote the manuscript. All authors read and approved the manuscript.

## Supplementary Material

Additional file 1 **Supplementary Figures and Table S1.** ΔD and ΔD_new_ in different (collapsed) protein interaction networks. **Figure S2:** Overlap between different yeast PINs. **Table S3:** Abundance of proteins in a PIN compared to the background. **Table S4:** Removal of most abundant proteins does not lead to a decrease in ΔD or ΔD_new_. **Table S5:** Removal of overrepresented functional categories does not lead to substantial decrease in ΔD or ΔD_new_. **Table S6:** ΔD, ΔD_new_ and the number of proteins in each age category for different age group definitions. **Figure S7:** Dn/Ds ratios for different age groups. **Table S8:** ΔD and ΔD_new_ in protein interaction networks for Dn/Ds categories. **Figure S9:** Duplication events can increase the number of interactions between proteins of a similar age. **Table S11:** Overlap of interaction partners of paralogs. **Table S12:** Interaction partners that are shared by paralogs more often have the same age. **Table S14:** Occurrence of duplication in one versus in both families in the LC Network. **Figure S15:** Cartoon explaining how protein families are incorporated in the Duplication Divergence model. **Figure S16:** Heatmap of ΔD for all parameter combinations we tried in the model. **Figure S17:** Heatmap of interaction densities between age groups in a network generated by the model for all parameter combinations we tried in the model. **Figure S18:** Heatmap of the fraction of edges that connects paralogs for all parameter combinations we tried in the model. **Figure S19:** ΔD for model networks before and after collapsing the network. **Figure S20:** Average relative overlap in interaction partners of paralogs. **Figure S21:** ΔD versus the average relative overlap in interaction partners between paralogs. **Figure S22:** the effect of removing randomly selected nodes or edges on ΔD and ΔD_new_. **Figure S23:** ΔD_new_ and new interaction densities between age groups in the original and collapsed protein interaction network. **Figure S24:** ΔD_new_ for model networks before and after collapsing the network. **Figure S26:** ΔD and ΔD_new_ in models networks using age group sizes similar to those in the data. **Figure S27:** The effect of including nodes without an age in the model on ΔD and the fraction of edges that connects paralogs. **Table S10: List of families with interparalog interactions in the LC network Table S13: List of family pairs that are connected by multiple edges in the LC network. Table S25: Table with all protein information used in this study.** Click here for file
