# Supplementary material for: Gene duplications contribute to the overrepresentation of interactions between proteins of a similar age
Source: BMC Evol Biol. 2012 Jun 25;12:99. doi: 10.1186/1471-2148-12-99 (PMC3457867; doi:10.1186/1471-2148-12-99)
Supplement: Additional file 1 — Supplementary Figures and Table S1. ΔD and ΔDnew in different (collapsed) protein interaction networks. Figure S2: Overlap between different yeast PINs. Table S3: Abundance of proteins in a PIN compared to the background. Table S4: Removal of most abundant proteins does not lead to a decrease in ΔD or ΔDnew. Table S5: Removal of overrepresented functional categories does not lead to substantial decrease in ΔD or ΔDnew. Table S6: ΔD, ΔDnew and the number of proteins in each age category for different age group definitions. Figure S7: Dn/Ds ratios for different age groups. Table S8: ΔD and ΔDnew in protein interaction networks for Dn/Ds categories. Figure S9: Duplication events can increase the number of interactions between proteins of a similar age. Table S11: Overlap of interaction partners of paralogs. Table S12: Interaction partners that are shared by paralogs more often have the same age. Table S14: Occurrence of duplication in one versus in both families in the LC Network. Figure S15: Cartoon explaining how protein families are incorporated in the Duplication Divergence model. Figure S16: Heatmap of ΔD for all parameter combinations we tried in the model. Figure S17: Heatmap of interaction densities between age groups in a network generated by the model for all parameter combinations we tried in the model. Figure S18: Heatmap of the fraction of edges that connects paralogs for all parameter combinations we tried in the model. Figure S19: ΔD for model networks before and after collapsing the network. Figure S20: Average relative overlap in interaction partners of paralogs. Figure S21: ΔD versus the average relative overlap in interaction partners between paralogs. Figure S22: the effect of removing randomly selected nodes or edges on ΔD and ΔDnew. Figure S23: ΔDnew and new interaction densities between age groups in the original and collapsed protein interaction network. Figure S24: ΔDnew for model networks before and after collapsing the network. Figure S26: ΔD [file 1471-2148-12-99-S1.pdf]

Table S1 ΔD and ΔD_{new} in different (collapsed) protein interaction networks

Network	Number of nodes	Number of edges	ΔD	ΔD_{new}
LC	3268	12058	0.51	0.35
edges between paralogs removed	3228	11628	0.38	0.2
multiple edges between families removed	1403	4933	0.12**	0.23
HTP	2488	6766	0.63	0.51
edges between paralogs removed	2415	6381	0.44	0.32
multiple edges between families removed	1105	3028	-0.07**	0.09
Y2H	1966	2705	0.6	0.44
edges between paralogs removed	1893	2609	0.36	0.21
multiple edges between families removed	872	1370	0.21*	0.13*
TAP	2390	16127	0.48	0.56
edges between paralogs removed	2316	15564	0.38	0.48
multiple edges between families removed	1061	6527	0.02**	-0.04**

* more than 1% of 100000 randomized networks have a ΔD value equal to or higher than this value (P value > 0.01)
** more than 10% of 100000 randomized networks have a ΔD value equal to or higher than this value (P value > 0.1)

A

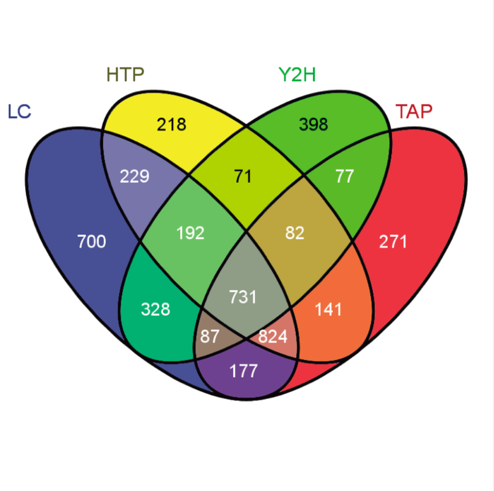

B

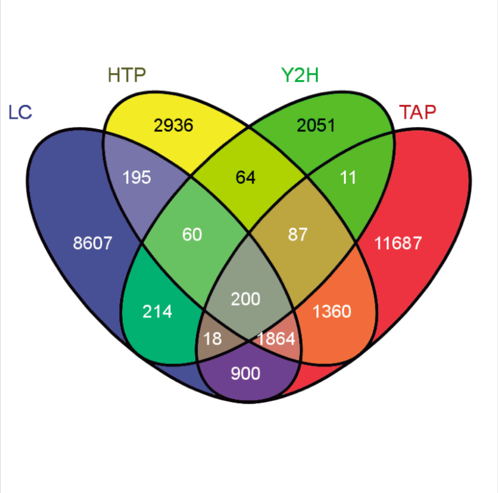

Figure S2: Overlap between different yeast PINs
A. Overlap in terms of proteins
B. Overlap in terms of interactions

Table S3: Abundance of proteins in a PIN compared to the background

	Proteins in the network		Proteins outside the network		
Network	Number of proteins	Mean ln(abundance)	Number of proteins	Mean ln(abundance)	P-value Mann Whitney test
LC	2432	7.84	1436	7.77	0.034
HTP	2029	7.97	1839	7.65	1.8e-13*
Y2H	1431	7.8	2437	7.82	0.27
TAP	1982	8.17	1886	7.44	9.12e-48*

We use abundance data that was provided in the Supplementary Material of Ghaemmaghami et al. [25] that reports protein abundances for 3868 *S. cerevisiae* ORFs. P-values marked with an * are below 0.01.

Table S4: removal of most abundant proteins does not lead to a decrease in ΔD or ΔD_{new}

	N	mean ln(abundance)	ΔD	ΔD_{new}	Number of edges in the network
LC	0	7.84	0.51	0.35	12058
	10	7.82	0.50	0.34	11661
	50	7.74	0.52	0.36	11237
	100	7.67	0.52	0.36	10910
	500	7.25	0.56	0.38	7958
	1000	6.77	0.54	0.32	4744
HTP	0	7.97	0.63	0.51	6766
	10	7.94	0.64	0.51	6565
	50	7.87	0.67	0.54	5982
	100	7.80	0.72	0.56	5500
	500	7.31	0.85	0.65	3132
	1000	6.69	0.77	0.63	1263
Y2H	0	7.80	0.60	0.44	2705
	10	7.77	0.61	0.45	2688
	50	7.66	0.62	0.47	2608
	100	7.56	0.63	0.49	2494
	500	6.97	0.61	0.4	1437
	1000	5.93	0.53	0.45	473
TAP	0	8.17	0.48	0.56	16127
	10	8.14	0.47	0.56	15855
	50	8.06	0.46	0.55	14617
	100	7.97	0.46	0.54	13471
	500	7.44	0.56	0.6	7501
	1000	6.84	0.60	0.68	3663

We use abundance data that was provided in the Supplementary Material of Ghaemmaghami et al. [25]. Protein abundances are reported for 3868 *S. cerevisiae* ORFs.

Table S5: Removal of overrepresented functional categories does not lead to substantial decrease in ΔD or ΔD_{new}.

Most eggNOG families have been assigned one or more functional categories named by a letter of the alphabet. For each network and each functional category, we count the number of proteins in the network that belong to a family assigned to that category and compare this number to the background of all yeast proteins. Functional categories named in the table below are significantly overrepresented in the network (P-value < 0.01, hypergeometric test).

To test whether ΔD or ΔD_{new} strongly depends on certain abundant functional categories we remove the overrepresented categories from the network and recalculate ΔD and ΔD_{new}, stated in the table below.

Network	Network size: nodes	Network size: edges	ΔD	ΔD_{new}	Fraction of edges connecting paralogs	Overrepresented categories (P<0.001)
LC	3268	12058	0.51	0.35	0.06	A, B, D, K, L, O, U, T, Z
HTP	2488	6766	0.63	0.51	0.09	A, B, D, K, L, R, T, Z
Y2H	1966	2705	0.6	0.44	0.06	D, K, O, U, T
TAP	2390	16127	0.48	0.56	0.05	A, B, D, K, J, O, U, Z

Removing proteins from families belonging to overrepresented functional categories entails removing a substantial number of nodes and edges. This does not lead to a decrease in ΔD or ΔD_{new}, rather these values are higher for these networks. This is striking because removal of random nodes leads to a decrease in ΔD and does not affect ΔD_{new} (Figure S22). In the reduced networks, the fraction of edges that connects paralogs is 1.5 to 3 fold higher than that of the original, unfiltered networks, which is reflected in both ΔD and ΔD_{new}. In the LC and TAP network most interparalog edges connect members from two families: COG2319, a family of proteins that contain the extremely promiscuous WD40 repeat (26 interparalog edges in LC and 79 interparalog edges in TAP) and COG0724, a family of RNA binding proteins that contain the RRM domain (20 interparalog edges in LC and 34 interparalog edges in TAP). In the reduced HTP network, there are 81 edges connecting members from COG0638, a family of alpha and beta subunits of the 20S proteasome.

Network	Network size: nodes	Network size: edges	ΔD	ΔD_{new}	Fraction of edges connecting paralogs
LC	1559	3482	0.69	0.48	0.09
HTP	1249	2236	0.71	0.66	0.15
Y2H	1199	1357	0.56	0.47	0.1
TAP	1263	2940	0.6	0.66	0.19

Table S6: **ΔD, ΔD_{new} and the number of proteins in each age category for different age group definitions**

Age group definition	Number of proteins in network			
	LC	HTP	Y2H	TAP
No age (not assigned to a COG/NOG)	792	579	523	547
**Age in main manuscript, ΔD**	**0.51**	**0.63**	**0.6**	**0.48**
**ΔD_{new}**	**0.35**	**0.51**	**0.44**	**0.56**
1: homologs in Bacteria and Archaea	1068	879	605	856
2: homologs in Bacteria or Archaea (not both)	299	234	190	276
3: homologs in Eukaryotes only	435	328	226	321
4: homologs in Fungi only	674	468	422	390
**Age 3, ΔD**	**0.6**	**0.72**	**0.74**	**0.62**
**ΔD_{new}**	**0.39**	**0.62**	**0.35**	**0.9**
1: homologs in Bacteria and/or Archaea	1367	1113	795	1132
2: homologs in Eukaryotes only	435	328	226	321
3: homologs in Fungi only	674	449	422	390
**Age 4, ΔD**	**0.76**	**0.86**	**0.92**	**0.67**
**ΔD_{new}**	**0.48**	**0.59**	**0.77**	**0.63**
1: homologs in Bacteria	1252	1020	720	987
2: homologs in Archaea only	115	93	75	145
3: homologs in Eukaryotes only	435	328	226	321
4: homologs in Fungi only	674	468	422	390
**Age 5, ΔD**	**0.79**	**1.03**	**0.85**	**1.06**
**ΔD_{new}**	**0.62**	**1.1**	**0.64**	**1.38**
1: homologs in Bacteria and/or Archaea	1367	1113	795	1132
2: homologs in Eukaryotes other than Ophistokonts	416	314	217	308
3: homologs in Ophistokonts other than Fungi	19	14	9	13
4: homologs in Fungi other than Ascomycetes	114	71	59	59
5: homologs in Ascomycetes	560	397	363	331
**Age 6, ΔD**	**0.7**	**0.88**	**0.78**	**0.85**
**ΔD_{new}**	**0.51**	**0.82**	**0.66**	**0.97**
1: homologs in Bacteria	1252	1020	720	987
2: homologs in Archaea only	115	93	75	145
3: homologs in Eukaryotes other than Ophistokonts	416	314	217	308
4: homologs in Ophistokonts other than Fungi	19	14	9	13
5: homologs in Fungi other than Ascomycetes	114	71	59	59
6: homologs in Ascomycetes	560	397	363	331

Families in the AE/BE category, with homologs in Bacteria and Eukaryotes or Archaea and Eukaryotes, are considered to be younger than families in the ABE (homologs in all three

Kingdoms) category. This means we assume that Archaea and Eukaryotes share a common ancestor and proteins with homologs in Bacteria only result from an endosymbiosis event leading to the mitochondrion. Loss of the protein in the ancestor of Archaea would give the same presence/absence pattern and proteins that are in the AE/BE category may have been present in the Last Universal Common Ancestor, but lost in either Archaea or Bacteria. We calculate ΔD as well as our alternative measure ΔD_{new} for the 4 different PINs, lumping the ABE and AE/BE categories into one ('age 3'), or, being more stringent with respect to the assumption that Archaea and Eukaryotes share a common ancestor, we consider any family with a homolog in Bacteria older than a family with a homolog in Archaea ('age 4') and find that the positive ΔD is not caused by these specific assumptions. Moreover, we use more fine-grained categories with respect to younger proteins, separating those families with a homog in only Ascomycota from the Fungal specific families and those with homologs in only Ophistokont from the Eukaryotic specific families, with ('age 6') and without ('age 5') assumptions regarding a shared ancestor of Archaea and Eukaryotes.

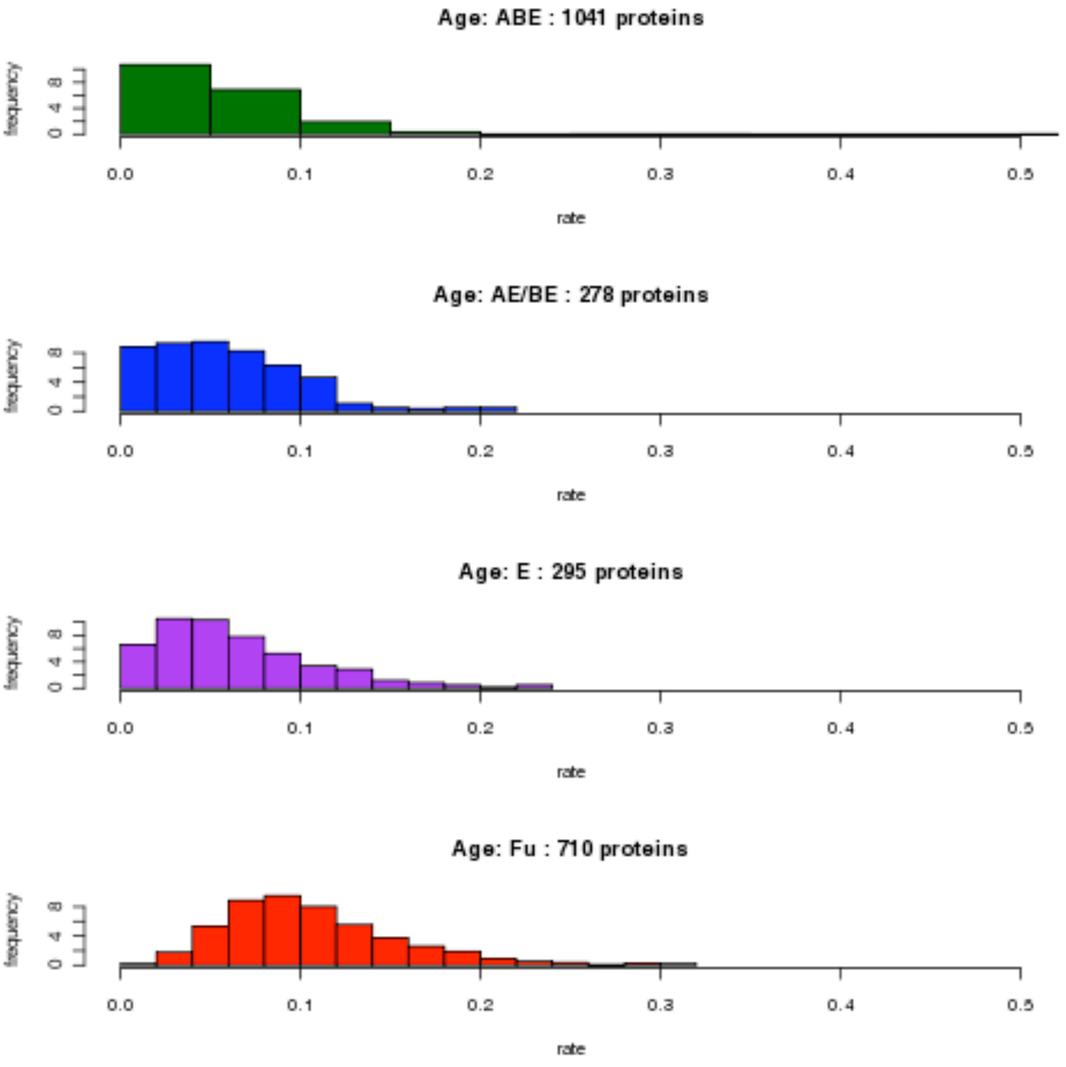

Figure S7: Dn/Ds ratios for different age groups
The frequency distribution of Dn/Ds ratios per age group shows faster sequence evolution for younger proteins than for older proteins.

Table S8: ΔD in protein interaction networks for Dn/Ds categories

ΔD for rate and age categories

network	ΔD rates	ΔD age	ΔD_{new} rates	ΔD_{new} age
LC	-0.05	0.54	0.24	0.33
HTP	-0.07	0.67	0.29	0.58
Y2H	0.07	0.67	0.13	0.5
TAP	0.25	0.51	0.37	0.51

N.B. ΔD and ΔD_{new} calculated over only those proteins that have a Dn/Ds value and are assigned to an age group

Number of proteins in each Dn/Ds bin: bins were chosen such that the number of proteins in each bins is similar to those observed for the real age categories (see Table S6)

	Number of proteins in network			
	LC	HTP	Y2H	TAP
Bin 1: Dn/Ds <= 0.06	699	575	412	595
Bin 2: 0.06 < Dn/Ds <= 0.08	203	146	127	137
Bin 3: 0.08 < Dn/Ds <= 0.1	199	135	104	122
Bin 4: Dn/Ds > 0.1	321	236	194	175
Total:	1422	1092	837	1029

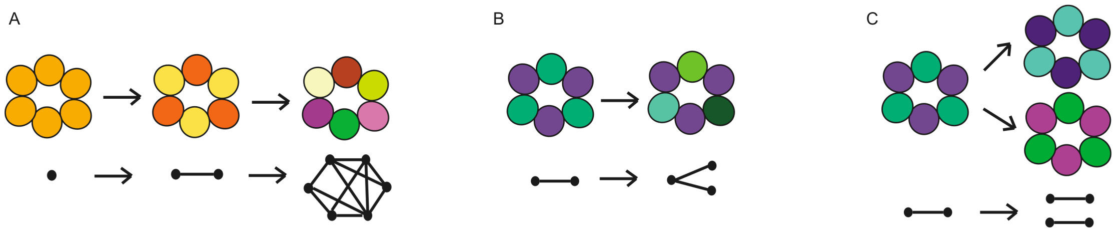

Figure S9: Duplication events can increase the number of interactions between proteins of a similar age

Ovals represent proteins. Multiple copies of the same protein are in the same color.
A. Growth of a functional module by incorporating duplicates of subunits. Different colours indicate different proteins that belong to the same family.
B. Conservation of an ancestral interaction in both paralogs. Ovals represent proteins. Purple ovals represent proteins that belong to one family and green ovals represent proteins that belong another family. Different shades of a colour indicate different proteins that belong to the same family. Edges connecting these two families overlap.
C. Co-duplication. Purple ovals represent proteins that belong to one family and green ovals represent proteins that belong another family. Different shades of a colour indicate different proteins that belong to the same family. Edges connecting these two families do not overlap.

Table S10 is in a separate file.

Table S11. Overlap of interaction partners of paralogs

	Number of pairs	Number of pairs with no overlap (fraction)	Mean overlap	Mean relative overlap
Paralogs	11559	9517 (0.82)	0.48	0.084
Non paralogs, of the same age	923966	875515 (0.95)	0.074	0.026
Non paralogs	3052491	2908418 (0.95)	0.066	0.022

Each row of the table contains the mean overlap in interaction partners for different classes of protein pairs: pairs of homologous proteins ('Paralogs'), pairs of proteins that are not homologous, but do have the same age ('Non paralogs, of the same age') and pairs of proteins that are not assigned to the same family ('Non paralogs').
The mean overlap is the average number of interaction partners shared by protein pairs of each category. The relative overlap is calculated by dividing the absolute overlap by the maximum possible overlap, which is the degree of the protein with the lowest number of interaction partners: $relative\_overlap_{x,y} = overlap_{x,y} / \min(degree_x, degree_y)$. We compare the average relative overlap in interaction partners for different categories of protein pairs. A Mann Whitney test comparing the relative overlap interaction partners of Paralogs vs. Non paralogs of the same age shows that paralogs share significantly more interaction partners than non paralogs of the same age (P ~ 0.0).

Table S12. Interaction partners that are shared by paralogs more often have the same age

	Total	Same age	Fraction same age
Partners shared by paralogs	4605	2167	0.47
Background (only paralog pairs that share at least one interaction partner)	43454	16166	0.37
Background (all paralog pairs)	166604	64815	0.38

For each pair of paralogs in the network, we compared the age of the interaction partners to the age of the paralogs. We find that interaction partners that are shared by paralogs (in total 4605 interactions partners for 11559 pairs) are more often of the same age, than interaction partners that are not shared between paralogs.

Table S13 is in a separate file

Table S14: Occurrence of duplication in one versus in both families in the LC network

LC network	No overlapping interactions	All inetractions are overlapping	Some interactions are overlapping, some are not	Total	Fraction overlapping
Family pairs of the same age	19	170	72	261	0.65
All family pairs	35	714	152	901	0.79
Family pairs of the same age, per age group:					
ABE	13	93	51	157	0.59
AE/BE	0	30	2	32	0.94
E	6	35	18	59	0.59
Fu	0	12	1	13	0.92

The most likely scenario (requiring the smallest number of evolutionary events) in which gene duplication generates additional edges between two families of similar age, is when a member *A* of one family duplicates and both daughters *A'* and *A"* keep the ancestral interaction with the protein *B* from the other family. The two edges representing these interactions overlap as both contain the protein *B*. On the other hand, if proteins from both families duplicate, the edges representing the interactions do not necessarily overlap: *A'* interacts with *B'* and *A"* interacts with *B"*.
For each family-pair that occurs multiple times in the network (i.e. multiple edges exist between members of these families), we calculate the fraction of protein-pairs that is overlapping. We find that for 80% of the families, all protein-pairs overlap (*A'-B* and *A"-B*, rightmost column in Table S14). If both families are of the same age, this fraction is much lower: 65%.

Results for the other networks are in separate tables below.

HTP network	No overlapping interactions	All interactions are overlapping	Some interactions are overlapping, some are not	Total	Fraction overlapping
Family pairs of the same age	16	102	28	146	0.7
All family pairs	34	350	70	454	0.77
Family pairs of the same age, per age group:					
ABE	13	59	24	96	0.61
AE/BE	2	25	1	28	0.89
E	1	15	3	19	0.79
Fu	0	3	0	3	1

Y2H network	No overlapping interactions	All interactions are overlapping	Some interactions are overlapping, some are not	Total	Fraction overlapping
Family pairs of the same age	4	25	2	31	0.81
All family pairs	6	77	6	89	0.87
Family pairs of the same age, per age group:					
ABE	4	12	2	18	0.67
AE/BE	0	1	0	1	1
E	0	5	0	5	1
Fu	0	7	0	7	1

TAP network	No overlapping interactions	All interactions are overlapping	Some interactions are overlapping, some are not	Total	Fraction overlapping
Family pairs of the same age	60	506	290	856	0.59
All family pairs	81	1350	571	2002	0.67
Family pairs of the same age, per age group:					
ABE	53	359	221	633	0.57
AE/BE	5	116	63	184	0.63
E	2	25	6	33	0.76
Fu	0	6	0	6	1

Figure S15: Cartoon explaining how protein families are incorporated in the Duplication Divergence model

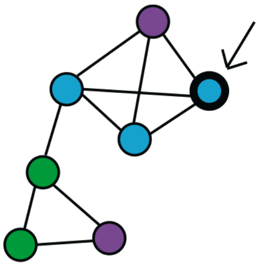

1
Select a random node

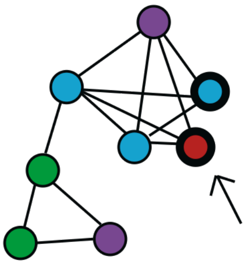

2
Duplicate node and its edges.
With a probability *a*:
one daughter is not recognized as a homolog, it starts a new family (new color: red) that is younger than the original family

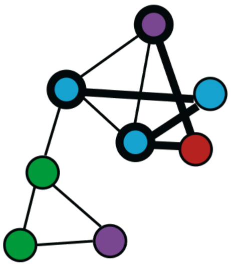

3
For each interaction partner: with a probability *q*: delete edge with one of the two daughters

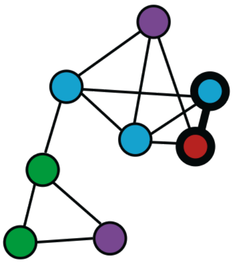

4
With a probability *p*: add a new edge connecting the two daughter nodes

Figure S16: Heatmap of ΔD for all parameter combinations we tried in the model. The left panel is a continuous heatmap of ΔD for all parameter combinations we tried in the model. Blue indicates high values of ΔD and yellow indicates low values. The right panel is the same heatmap in which each set of parameter conditions is assigned to a category: fitting the data (bright green), too high values (dark green) and too low values (red).

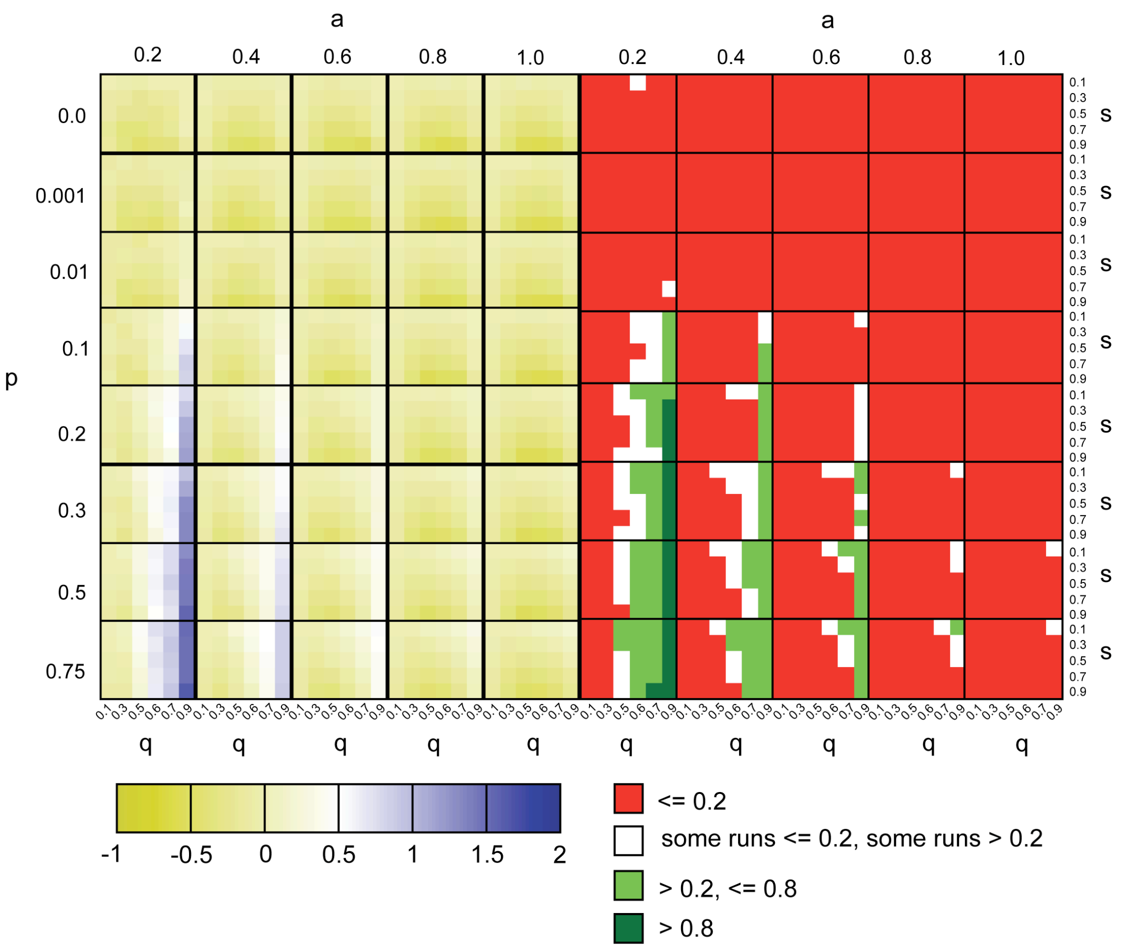

Figure S17: Heatmap of interaction densities between age groups in a network generated by the model

The network for which the interaction densities are shown was generated using default parameter settings (p=0.2, q=0.7, a=0.2, s=0.5) and has a ΔD value of 0.54. Dark purple squares along the diagonal indicate a strong overrepresenation of interactions between proteins of the same age, which in this case boils down to interactions between proteins of the same family.

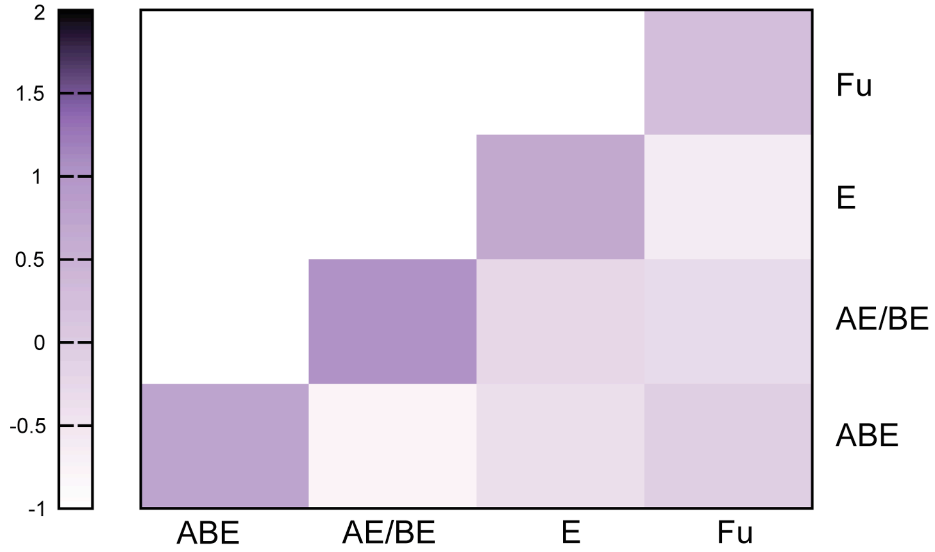

Figure S18: Heatmap of the fraction of edges that connects paralogs for all parameter combinations we tried in the model

The left panel is a continuous heatmap of the fraction of edges that connects paralogs for all parameter combinations we tried in the model. Blue indicates a high proportion of all edges connects members of the same family. The right panel is the same heatmap in which each set of parameter conditions is assigned to a category: fitting the data (bright green), too high values (dark green) and too low values (red).

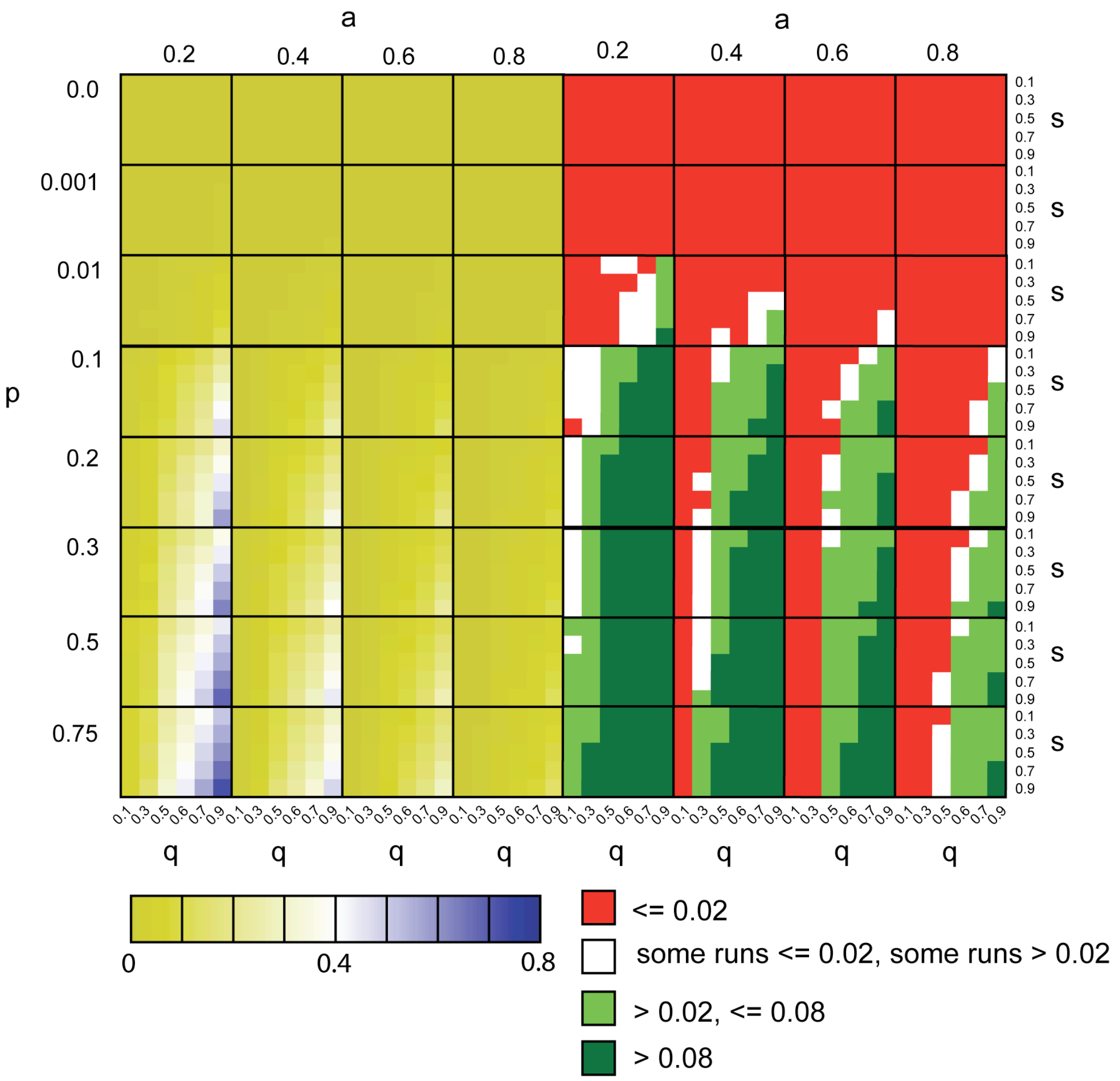

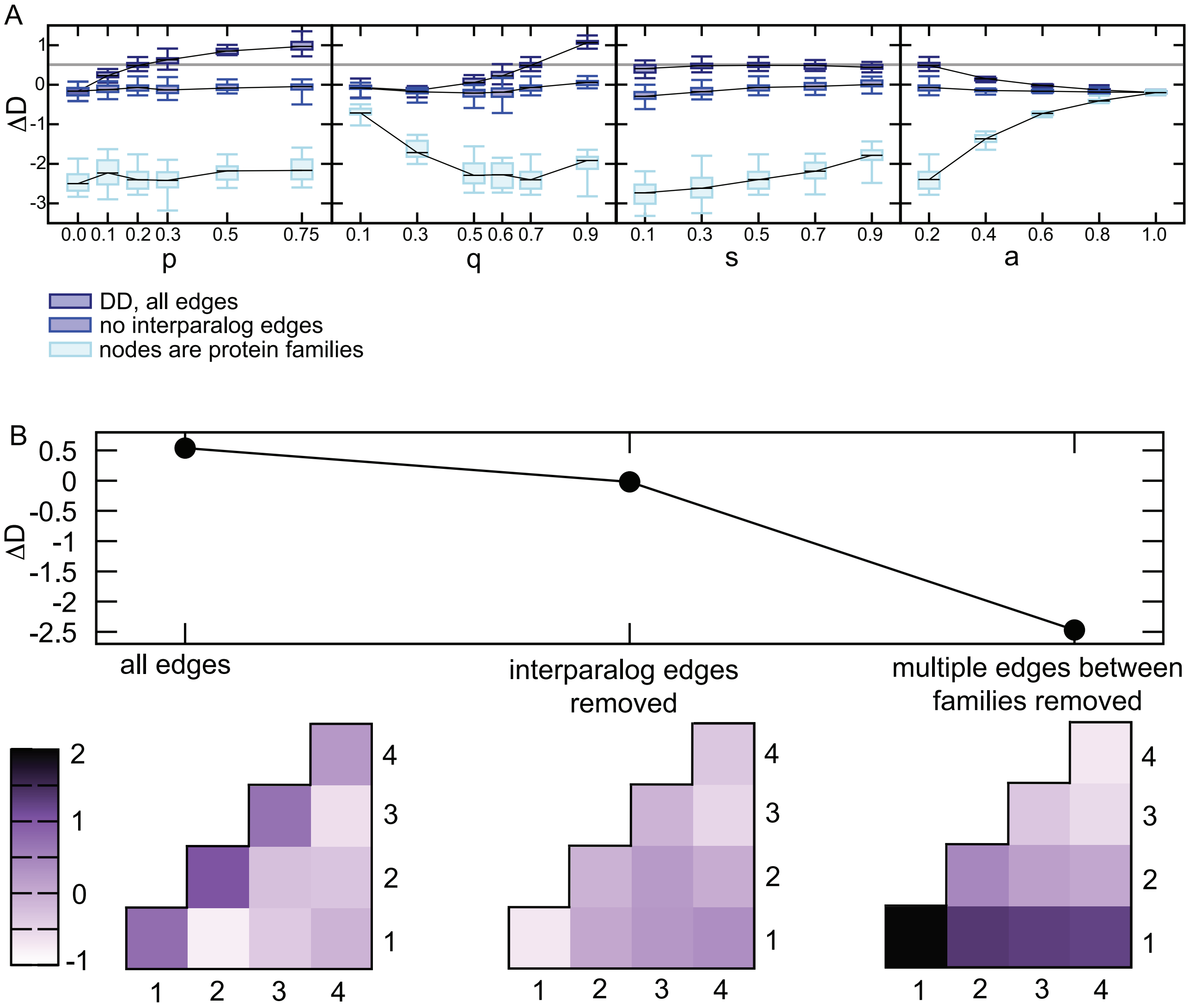

Figure S19: ΔD for model networks before and after collapsing the network.
As we did with real yeast PINs, we remove edges that result from duplication events from model networks and study the effect on ΔD.
A. ΔD for model networks in different parameter conditions: all edges, no interparalog edges and networks collapsed into networks of protein families. Default parameter conditions are p=0.2, q=0.7, s=0.5, a=0.2, each plot shows ΔD values when one of these parameters is varied while the others are kept at default values. The gray line is the ΔD value of the yeast LC PIN. Boxes show the .25 and .75 percentile of 20 runs, the error bars show the extreme values and the black line is the mean of 20 runs.
B. ΔD and interaction densities for a model network generated using default parameter settings (p=0.2, q=0.7, a=0.2, s=0.5): all edges, no interparalog edges and networks collapsed into networks of protein families.

In the data, removing interparalog edges from the network had little effect on ΔD. In the model networks, ΔD drops to zero after removing interparalog edges, indicating the positive ΔD value strongly depends on interactions between paralogs. If we collapse the network into a network of protein families rather than proteins we see a further decline in ΔD. This mainly reflects a bias in connectivity in the collapsed model networks: old families have a high degree. We do not observe this in real networks.

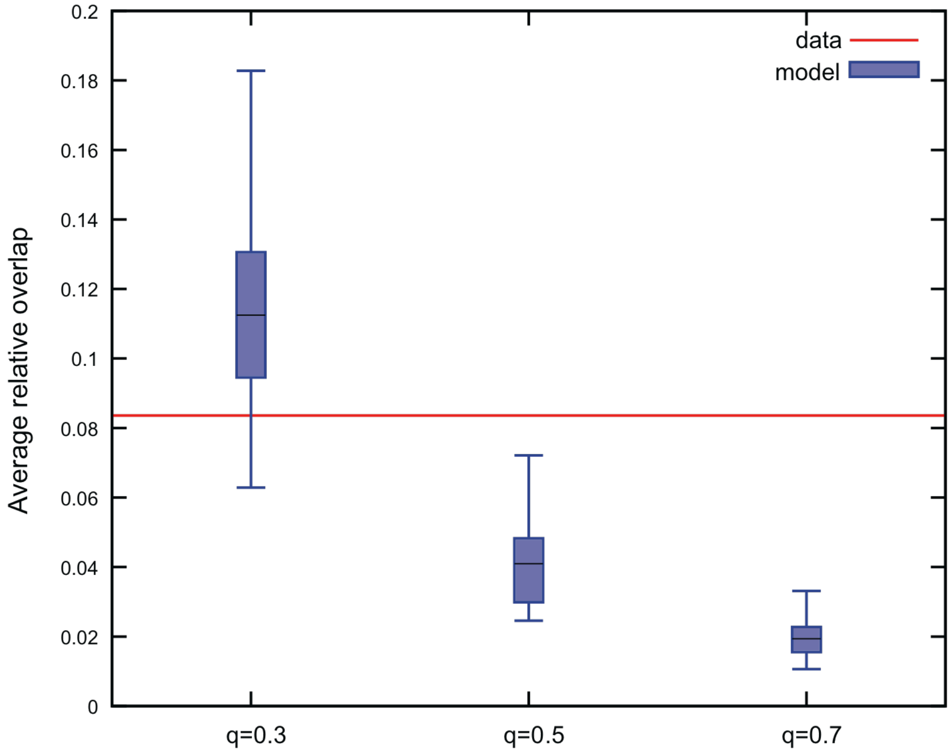

Figure S20: Average relative overlap in interaction partners of paralogs.
Boxplot with the average relative overlap in interaction partners of paralogs for different levels of divergence after duplication (different values of q). Average relative overlap is the overlap divided by the maximum overlap (see Table S11). Boxes show the .25 and .75 percentile of 20 runs, the error bars show the extreme values and the black line is the mean of 20 runs.

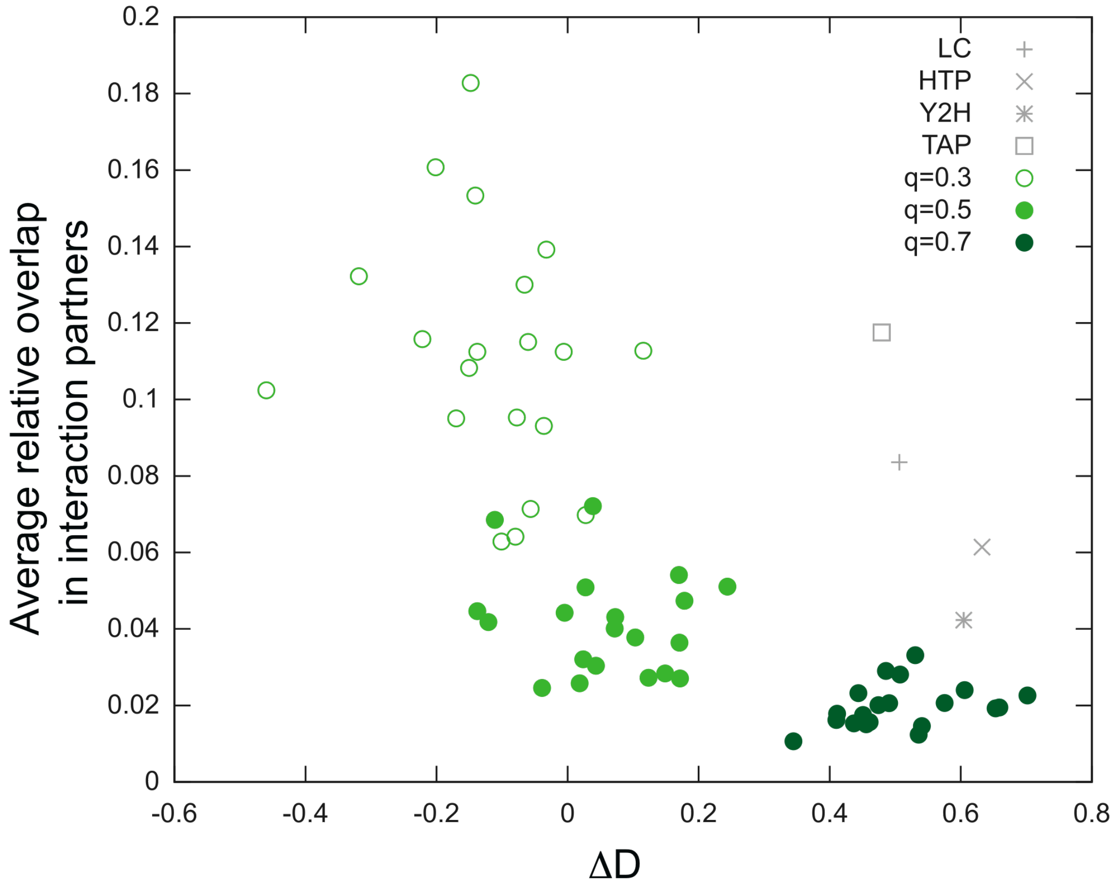

Figure S21: ΔD versus the average relative overlap in interaction partners between paralogs

Scatterplot of ΔD versus the average relative overlap in interaction partners between paralogs, for different levels of divergence after duplication (different values of q in green, p=0.2, a=0.2, s=0.5) and different yeast PINs (in grey). Average relative overlap is the overlap divided by the maximum overlap (see Table S11).

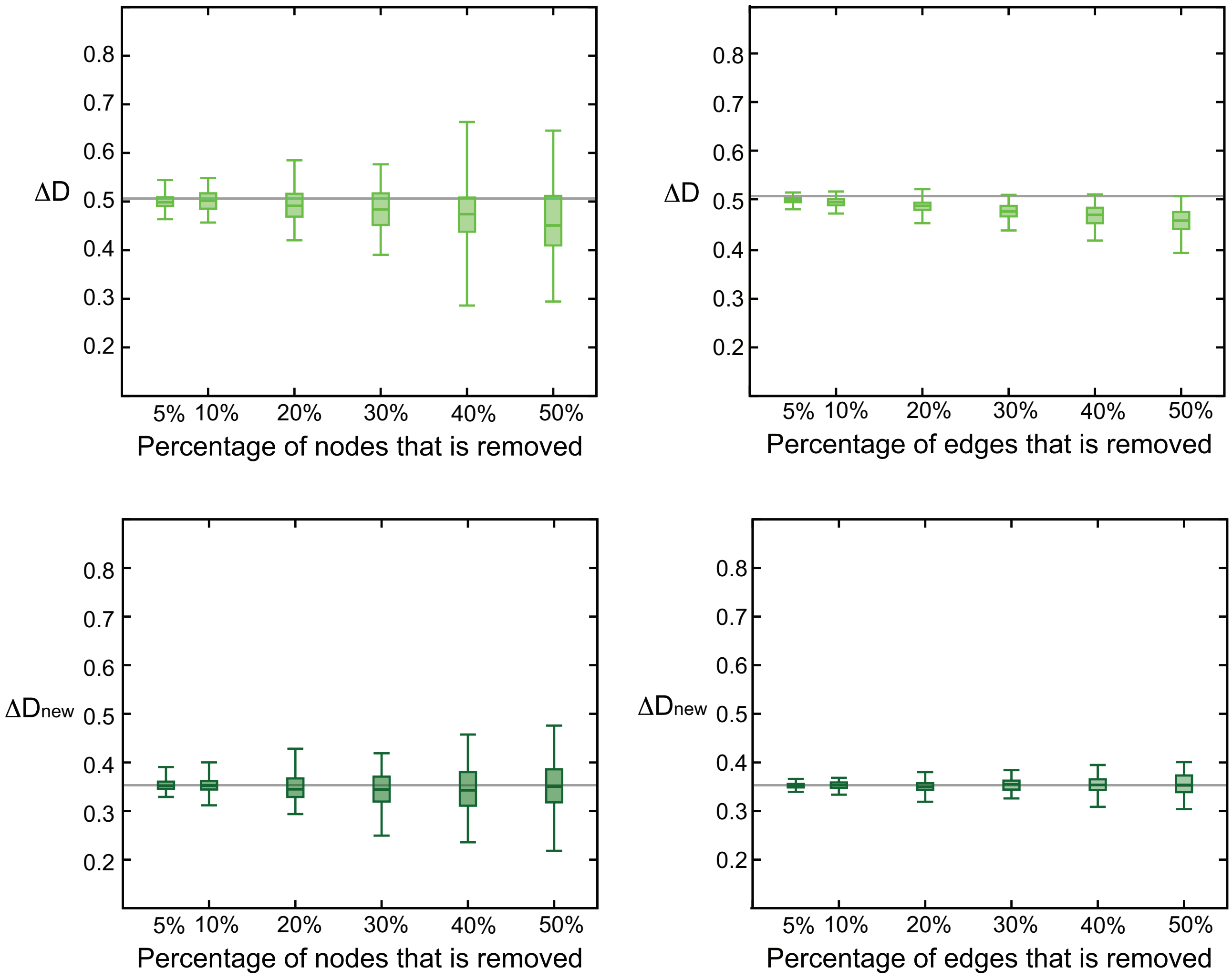

Figure S22: the effect of removing randomly selected nodes or edges on ΔD and ΔD_{new}.

We randomly selected a certain percentage (x-axes) of nodes (left panels) or edges (right panels) and removed them from the LC network. Boxes show the .25 and .75 percentile of ΔD (green, top panels) and ΔD_{new} (dark-green, bottom panels) values of 100 iterations, the error bars show the extreme values and the black line is the mean. ΔD decreases as more nodes or edges are removed from the network, whereas ΔD_{new} does not.

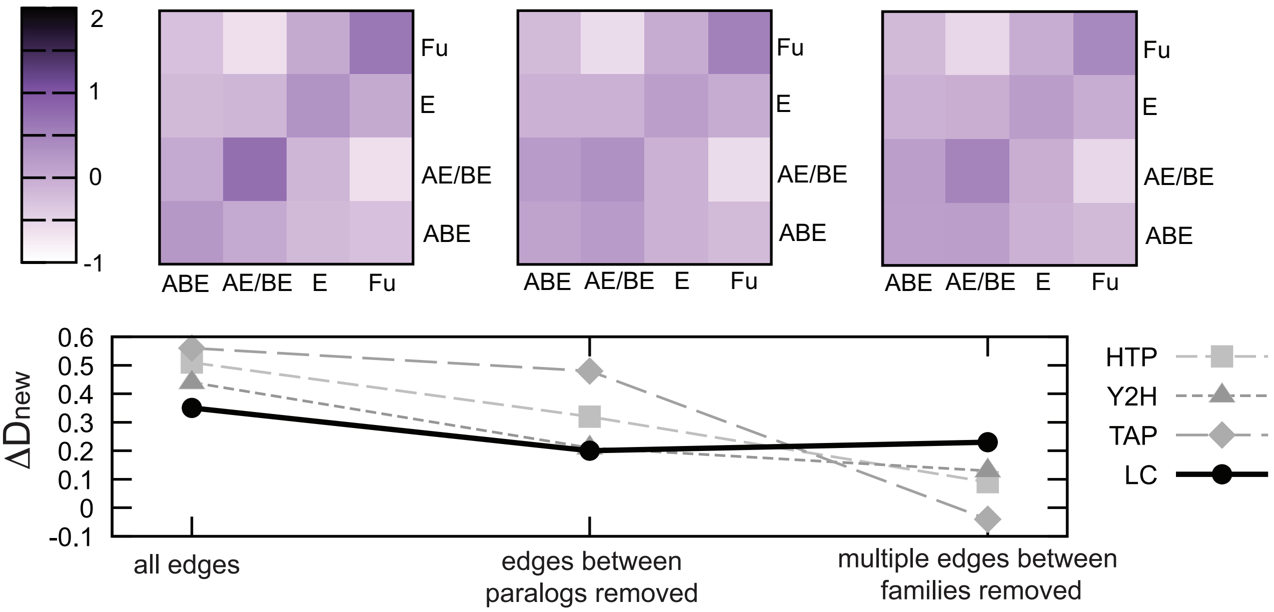

Figure S23: ΔD_{new} and new interaction densities between age groups in the original and collapsed protein interaction network.
We calculate interaction densities normalized by expected interaction densities and determine the gradient ΔD_{new} for 4 different *S. cerevisiae* protein intercation networks (see Materials and Methods in main text for more detail). If we remove all edges between paralogs, the ΔD_{new} value decreases (as should be expected as all these edges are between proteins of the same age). We then continue to remove all edges that are redundant on a protein family level, thus collapsing the network into a network where nodes are no longer proteins, but protein families. In the LC network (black line) ΔD_{new} is not decreased further, in the other networks (grey lines) it is (see also Table S1). Interaction densities (top) are only shown for the LC network.

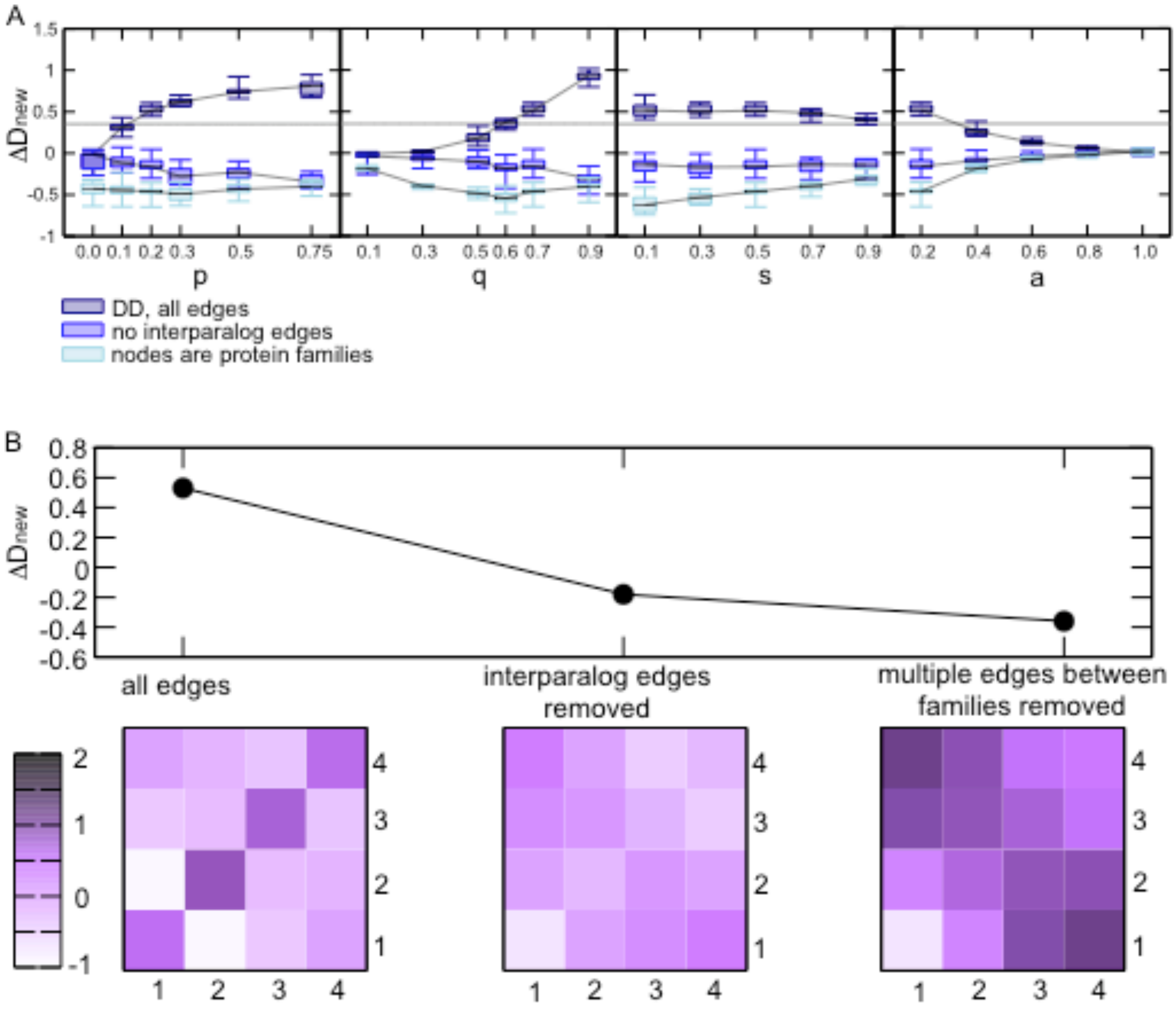

Figure S24: ΔD_{new} for model networks before and after collapsing the network.
As we did with real yeast PINs, we remove edges that result from duplication events from model networks and study the effect on ΔD_{new}.
A. ΔD_{new} for model networks in different parameter conditions: all edges, no interparalog edges and networks collapsed into networks of protein families. Default parameter conditions are p=0.2, q=0.7, s=0.5, a=0.2, each plot shows ΔD_{new} values when one of these parameters is varied while the others are kept at default values. The gray line is the ΔD_{new} value of the yeast LC PIN. Boxes show the .25 and .75 percentile of 20 runs, the error bars show the extreme values and the black line is the mean of 20 runs.
B. ΔD_{new} and interaction densities for a model network generated using default parameter settings (p=0.2, q=0.7, a=0.2, s=0.5): all edges, no interparalog edges and networks collapsed into networks of protein families.

Because ΔD_{new} does not depend on differences in connectivity between age groups we do not see a strong additional decline when we collapse the model network into families.

Table S25 is in a separate file

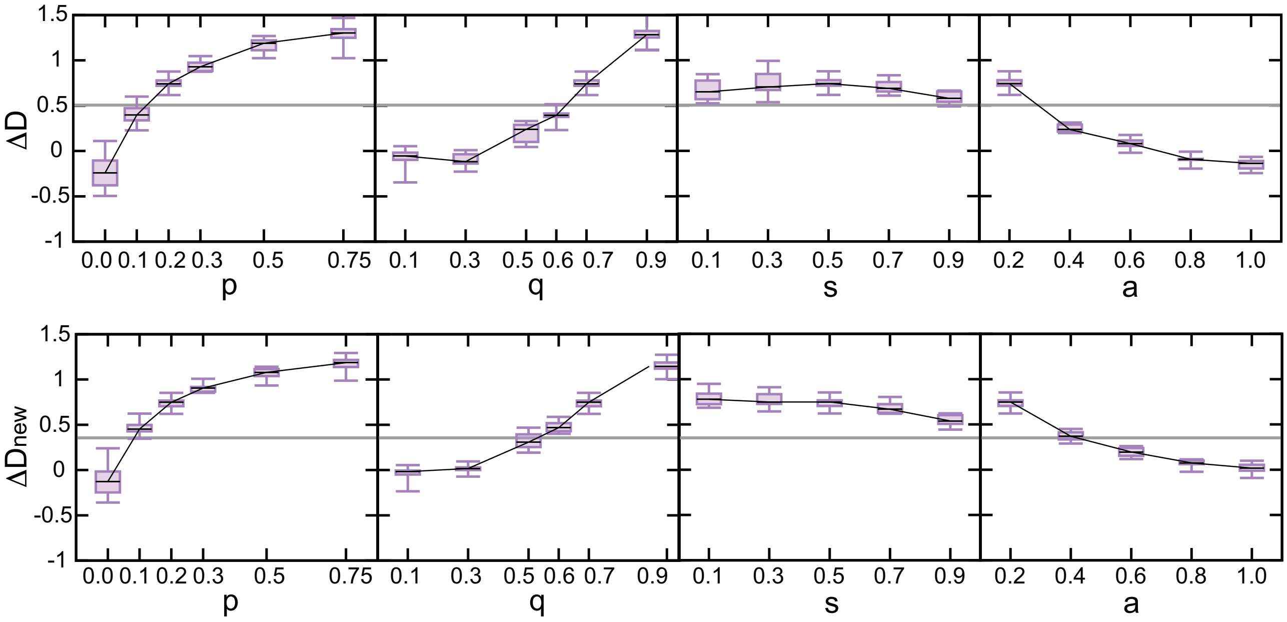

FigureS26: ΔD and ΔD_{new} in models networks using age group sizes similar to those in the data.

Default parameter conditions are p=0.2, q=0.7, s=0.5, a=0.2, each plot shows ΔD (top) and ΔD_{new} (bottom) values when one of these parameters is varied while the others are kept at default. The gray line is the ΔD resp. ΔD_{new} value of the yeast LC PIN. Boxes show the .25 and .75 percentile of 10 runs, the error bars show the extreme values and the black line is the mean of 10 runs. The size distribution of age groups does neither affect ΔD as this figure is very similar to Figure 3 in the main text, nor ΔD_{new} (similar to Figure S24)

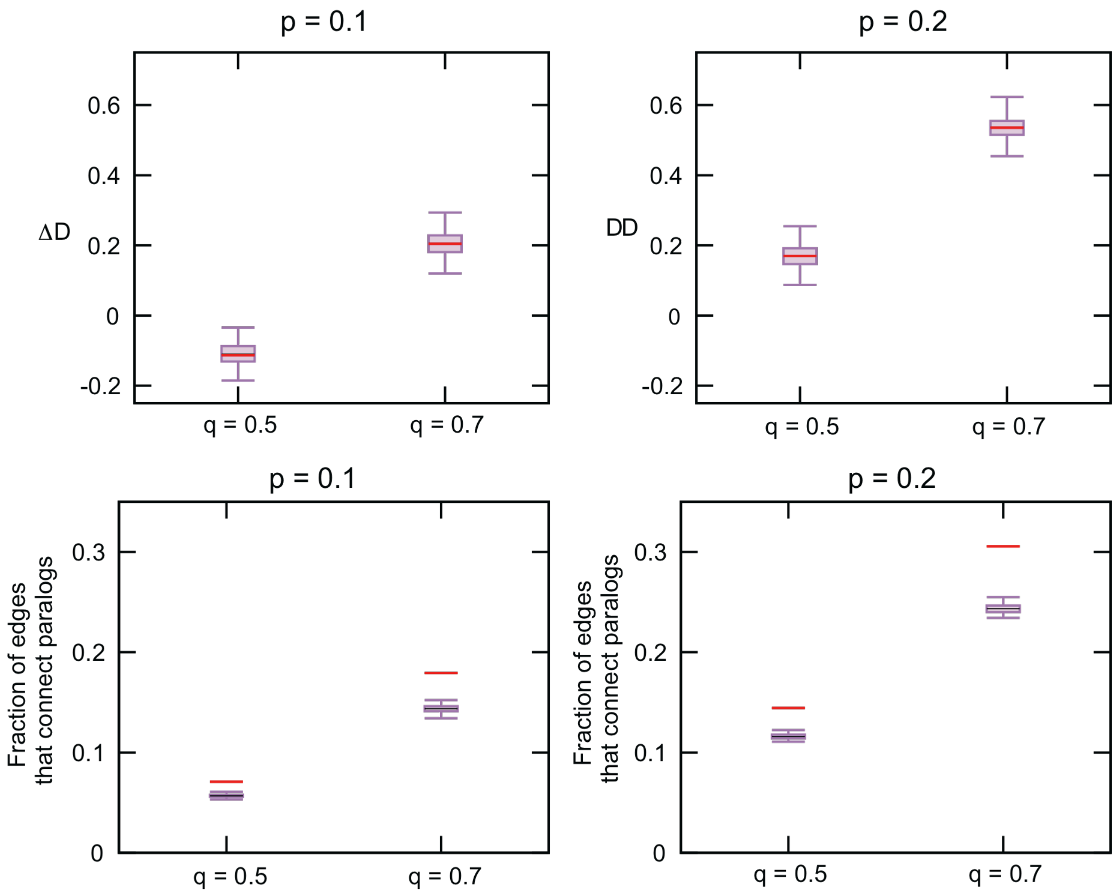

Figure S27: The effect of including nodes without an age in the model on ΔD and the fraction of edges that connects paralogs.

We take one of the 20 model networks for a given parameter setting (p is 0.1 or 0.2, q is 0.5 or 0.7, a is 0.2 and s is 0.5) and randomly select 600 nodes, of which we designate the age as unknown and calculate ΔD and the fraction of edges that connects paralogs for this network, for 100 times (purple boxplots, box corresponds to the .25-.75 percentiles). The red line is ΔD or fraction of edges that connects paralogs for this specific model network.
